# Optimization of Pinocembrin Biosynthesis in *Saccharomyces cerevisiae*

**DOI:** 10.1021/acssynbio.2c00425

**Published:** 2022-12-19

**Authors:** Marta Tous Mohedano, Jiwei Mao, Yun Chen

**Affiliations:** Department of Biology and Biological Engineering, Chalmers University of Technology, Göteborg SE41296, Sweden

**Keywords:** flavonoids, tolerance, byproduct, pathway optimization, yeast

## Abstract

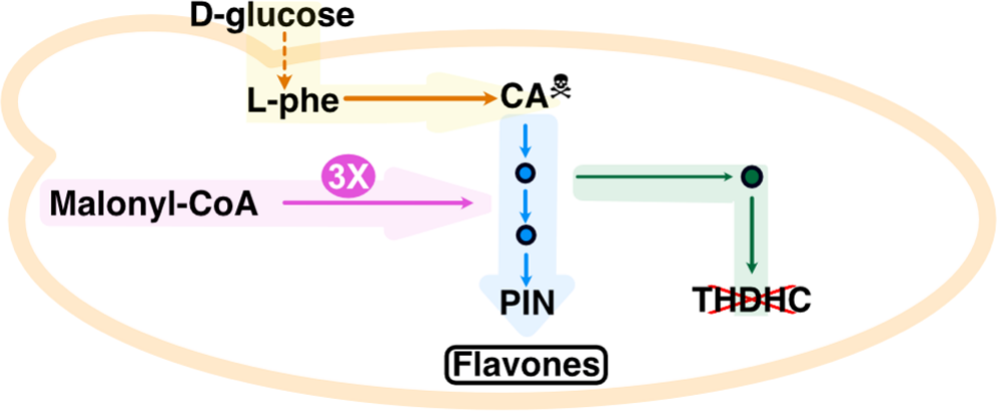

The flavonoid pinocembrin and its derivatives have gained increasing interest for their benefits on human health. While pinocembrin and its derivatives can be produced in engineered *Saccharomyces cerevisiae*, yields remain low. Here, we describe novel strategies for improved *de novo* biosynthesis of pinocembrin from glucose based on overcoming existing limitations in *S. cerevisiae.* First, we identified cinnamic acid as an inhibitor of pinocembrin synthesis. Second, by screening for more efficient enzymes and optimizing the expression of downstream genes, we reduced cinnamic acid accumulation. Third, we addressed other limiting factors by boosting the availability of the precursor malonyl-CoA, while eliminating the undesired byproduct 2′,4′,6′-trihydroxy dihydrochalcone. After optimizing cultivation conditions, 80 mg/L pinocembrin was obtained in a shake flask, the highest yield reported for *S. cerevisiae*. Finally, we demonstrated that pinocembrin-producing strains could be further engineered to generate 25 mg/L chrysin, another interesting flavone. The strains generated in this study will facilitate the production of flavonoids through the pinocembrin biosynthetic pathway.

## Introduction

Flavonoids are polyphenolic compounds found in vegetables, nuts, fruits, tea, seeds, and red wine. They possess antimicrobial, anti-inflammatory, cardioprotective, antioxidant, and antiaging bioactivity.^1,2^ Flavonoids can be obtained directly from plants, or they can be synthesized chemically; however, both processes present disadvantages. On the one hand, the low abundance of flavonoids in nature requires extraction from large amounts of plant material and complex purification processes.^3^ On the other hand, the structural complexity of flavonoids hampers bulk chemical synthesis, which often relies on toxic reagents, harsh operating conditions, and abundant waste. Microbial cell factories have been proposed as a sustainable means to meet the increasing demand for these natural plant products.^4,5^

**Figure 1:**
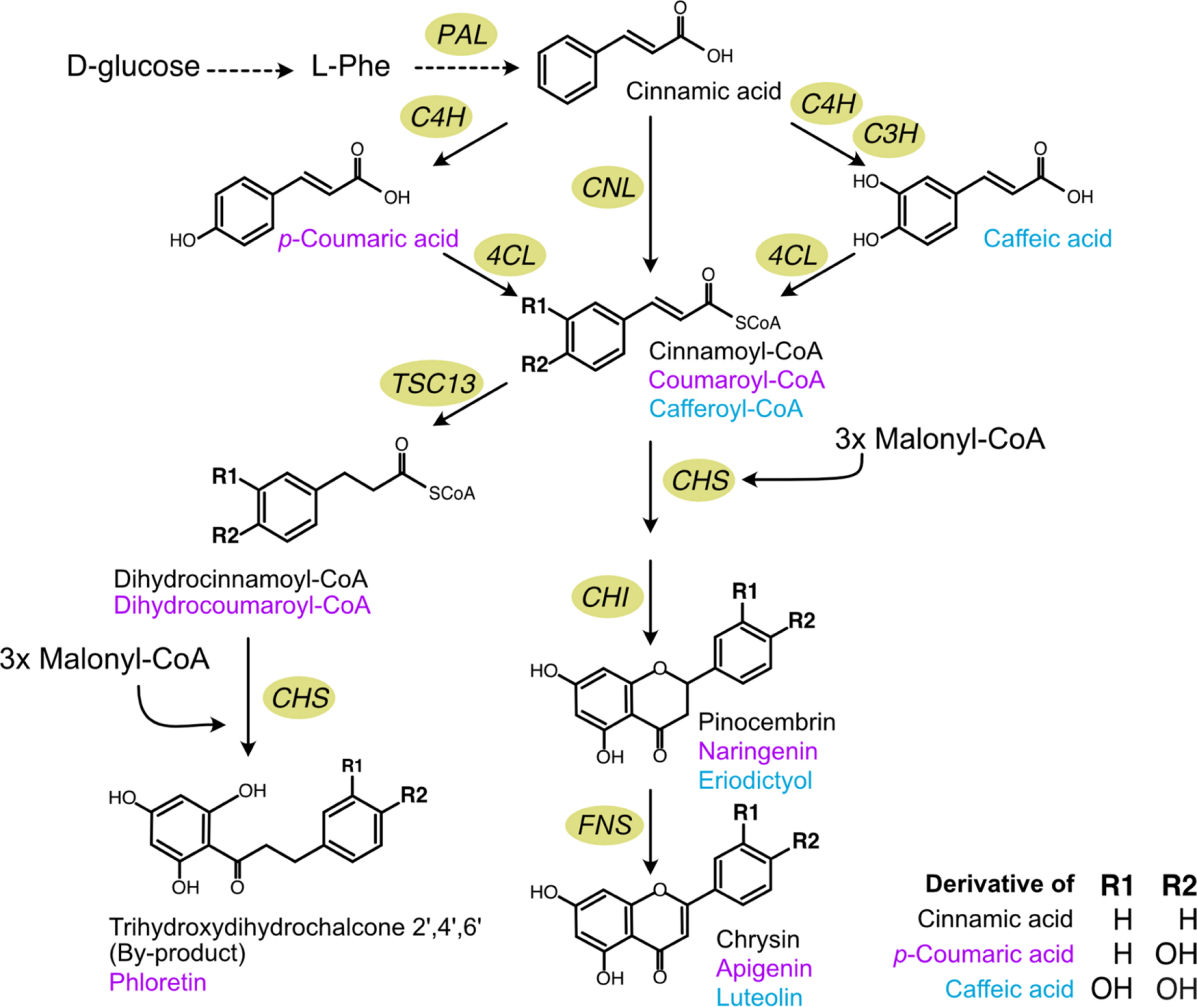
Schematic diagram of the pathway established in *S. cerevisiae* to produce pinocembrin, naringenin, and eriodictyol, as well as their derivatives. Solid lines represent a single step; dashed lines indicate multiple steps; purple font denotes derivatives of *p*-coumaric acid; blue font denotes derivatives of caffeic acid; black font denotes derivatives of cinnamic acid; l-phe, l-phenylalanine; PAL, phenylalanine ammonia lyase; CNL, cinnamate-CoA ligase; C4H, cinnamate-4-hydroxylase; C3H, *p*-coumarate-3-hydroxylase; 4CL, *p*-coumaroyl-CoA ligase; CHS, chalcone synthase; CHI, chalcone isomerase; TSC13, enoyl reductase; FNS, flavone synthase.

Over the last decade, flavonoid biosynthetic pathways have been engineered and optimized in several industrially relevant microorganisms, such as *Saccharomyces cerevisiae* and *Escherichia coli*.^6,7^ From the aromatic amino acid l-phenylalanine, different classes of flavonoids, for example, pinocembrin, naringenin, and eriodictyol can be obtained from three different intermediates: cinnamic acid (CA), *p*-coumaric acid (*p*-HCA), and caffeic acid, respectively (Figure 1). The production of naringenin has been studied extensively in different host organisms, including *E. coli*,^8^*Yarrowia lipolytica*,^9^ and *S. cerevisiae*,^10−12^ and has resulted in up to g/L of this product in bioreactors. In contrast, production of pinocembrin and its derivatives, such as chrysin, baicalein, and wogonin, has received less attention. Pinocembrin possesses antibacterial,^13^ cardioprotective,^14^ and neuroprotective^15^ properties, which make this compound a very attractive target for the pharmaceutical industry. Similarly, interesting activities have been reported for chrysin, baicalein, and wogonin.^16−20^*De novo* production of pinocembrin in *E. coli* was reported at 198 mg/L in 96-deep-well blocks and at 525.8 mg/L in bioreactors under fed-batch-like conditions.^21,22^ As a eukaryotic organism, *S. cerevisiae* can carry out post-translational modifications on plant-derived proteins and express type II P450 hydroxylases.^23^ Nevertheless, the production of pinocembrin and its derivatives in this organism has not been as successful as in other hosts, with maximum yields of 16.3 mg/L when fed on 1 mM CA.^24^ Similar low yields have been reported for chrysin (3.63 mg/L) and baicalein (4.69 mg/L).^25^ Therefore, there is a strong interest in improving the production of pinocembrin and its derivatives in *S. cerevisiae*.

The biosynthesis of pinocembrin begins with the conversion of l-phenylalanine to CA by phenylalanine ammonia lyase (PAL). Second, CA is converted to cinnamoyl-CoA by cinnamate-CoA ligase (CNL). Third, three molecules of malonyl-CoA are added sequentially to cinnamoyl-CoA by chalcone synthase (CHS) to produce trihydroxychalcone. Finally, chalcone isomerase (CHI) converts the last compound to pinocembrin^24,26^ (Figure 1). This pathway also generates 2′,4′,6′-trihydroxy dihydrochalcone (THDHC) as an undesired byproduct. THDHC was first detected together with phloretin when producing pinocembrin and naringenin in yeast, but the underlying enzymatic mechanism had not been elucidated.^27^ Lehka et al. discovered that the yeast’s native enoyl reductase Tsc13 could convert *p*-coumaroyl-CoA to *p*-dihydrocoumaroyl-CoA;^28^ however, no direct proof of cinnamoyl-CoA production exists (Figure 1).

In this study, we aimed to identify the limiting steps of pinocembrin production in *S. cerevisiae* and design metabolic engineering strategies to improve its *de novo* biosynthesis. First, we showed that CA hindered the production of pinocembrin, possibly due to its toxicity. Strains with an increased flux of aromatic amino acids produced 10 times less CA than *p*-HCA, which is a hydrolyzed product of CA. Next, to reduce the accumulation of CA, we applied the same strategy as reported earlier for the production of CA derivatives in yeast.^29,30^ This involved screening for more efficient enzymes and optimizing the expression of downstream genes. Increasing CA consumption improved pinocembrin production by 3-fold; however, this value was still lower compared to that achieved for other classes of flavonoids. Therefore, we increased the flux of another important precursor, malonyl-CoA, by introducing either a bacterial malonate assimilation pathway or a mutant acetyl-CoA carboxylase. Both strategies improved pinocembrin production. A third line of investigation revealed that THDHC was produced from cinnamoyl-CoA via a two-step reaction catalyzed by Tsc13 and a heterologous CHS. Moreover, when these side reactions were blocked, the flux toward pinocembrin increased. Finally, we optimized the cultivation conditions and attained 80 mg/L pinocembrin, which represented a 13-fold increase compared to the starting strain. We believe that our strains can be optimized to further improve pinocembrin production and serve as platforms for the biosynthesis of downstream flavonoids, such as chrysin, baicalein, baicalin, wogonin, and norwogonin.

## Results

### Cinnamic Acid Is a Potential Bottleneck for Pinocembrin Production

**Figure 2:**
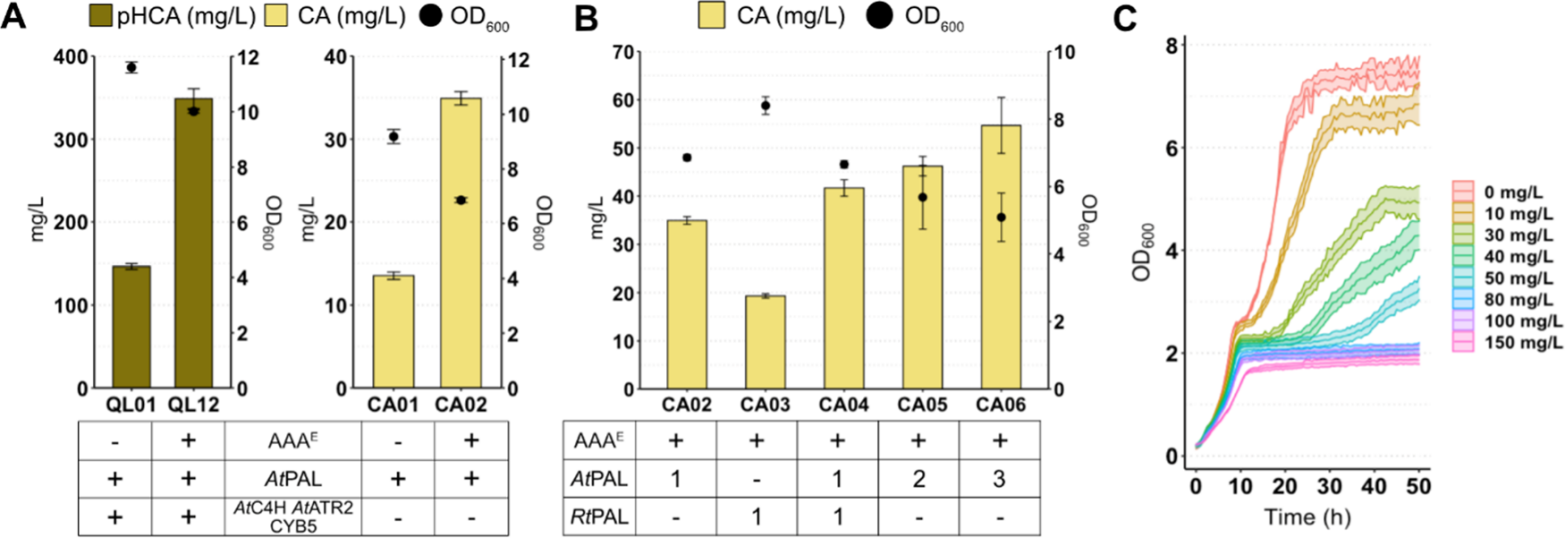
Toxicity of cinnamic acid in *S. cerevisiae*. (A) *p*-Coumaric acid and cinnamic acid production by engineered strains expressing the core *p*-coumaric acid and cinnamic acid synthetic genes, respectively. (B) Production of cinnamic acid by engineered strains expressing different phenylalanine ammonia lyase constructs at varying copy number. In (A) and (B), strains were cultivated in batch for 72 h at 30 °C using defined minimal medium supplemented with 30 g/L glucose. (C) Growth curves of IMX581 cultivated in 96-well plates in the presence of different concentrations of cinnamic acid. Growth was measured every 30 min. Data represent the mean of *n* = 3 independent biological samples, and error bars denote the standard deviation. *p*-HCA, *p*-coumaric acid; CA, cinnamic acid; OD_600_, optical density; AAA^E^, overexpression of Aro7^G141S^, Aro4^K229L^, ARO1/2/3, PHA2, EcAroL from *E. coli*; AtPAL, phenylalanine ammonia lyase from *A. thaliana*; RtPAL phenylalanine ammonia lyase from *Rhodotorula toruloides*; AtC4H, cinnamic acid hydroxylase from *A. thaliana*; AtATR, cytochrome P450 reductase from *A. thaliana*; CYB5, yeast native cytochrome b5.

CA is the first intermediate in the biosynthesis of pinocembrin from l-phenylalanine. To produce CA in *S. cerevisiae*, PAL from *Arabidopsis thaliana* (*At*PAL) was introduced into the genome. This enzyme, together with *A. thaliana* cinnamic acid hydroxylase (*At*C4H), was previously reported to efficiently generate *p*-HCA in *S. cerevisiae*.^31^ When (i) the allosteric regulation of 3-deoxy-d-arabinoheptulosonate 7-phosphate (DAHP) synthase and chorismite mutase was alleviated, (ii) *PHA2*, *ARO1*, *ARO2*, and *ARO3* were overexpressed, and (iii) the heterologous shikimate kinase AroL from *E. coli* was introduced, the resultant AAA^E^ background strain led to significantly increased flux in aromatic amino acid biosynthesis in yeast.^31,32^ Therefore, *At*PAL was expressed in a wild-type and an engineered AAA^E^ background, resulting in two new strains, CA01 and CA02, which produced 14 and 35 mg/L CA, respectively (Figure 2A). A comparison of *p*-HCA and CA production in the wild-type (QL01 vs CA01) and AAA^E^ (QL12 vs CA02) backgrounds, revealed almost 10 times lower CA than *p*-HCA yields, irrespective of strain background (Figure 2A). The only difference was related to the end products (Figure 1) generated by *At*C4H upon conversion of all CA to *p*-HCA in strains QL01 and QL12 (Figure S1). The two CA strains (CA01 and CA02) showed 20 and 32% lower final OD_600_ when compared to the corresponding *p*-HCA-producing strains (QL01 and QL12) (Figure 2A). The low yield of CA compared to *p*-HCA, as well as the growth difference between CA- and *p*-HCA-producing strains pointed to CA as a bottleneck in pinocembrin production from glucose.

Based on the higher CA production in strain CA02 compared to CA01, we chose the former for further studies. To increase CA production, another commonly used ammonia lyase, this one from *R. toruloides* (*Rt*PAL), was tested in strain CA03. *Rt*PAL halved CA output (Figure 2B), suggesting that *At*PAL worked better than Rt*PAL*. Therefore, we investigated if a strain with a combination of both PAL enzymes performed better or if additional copies of *At*PAL could increase CA production. Of the resulting strains (CA04, CA05, and CA06), CA06 produced up to 60 mg/L (Figure 2B), but more copies of *PAL* slowed down growth. These results suggested that CA accumulation was stressful for the cell and impaired growth.

Given that CA is potentially toxic to microbial cells, we evaluated the growth of a wild-type strain, IMX581,^33^ in the presence of 0–150 mg/L CA (Figure 2C). We found that growth was halved when the strain was cultivated with more than 50 mg/L CA. Such growth reduction was more dramatic than previously observed under pH-buffered conditions,^29,34^ suggesting a role of pH in CA toxicity. To circumvent this problem, high-density bioconversion has been proposed.^29^ This measure, however, augments population heterogeneity^34^ and, thus, compromises production performance. Instead, we focused on increasing the downstream CA flux, thereby minimizing CA accumulation and inhibition of pinocembrin biosynthesis.

### Optimized Conversion of Cinnamic Acid to Pinocembrin

**Figure 3:**
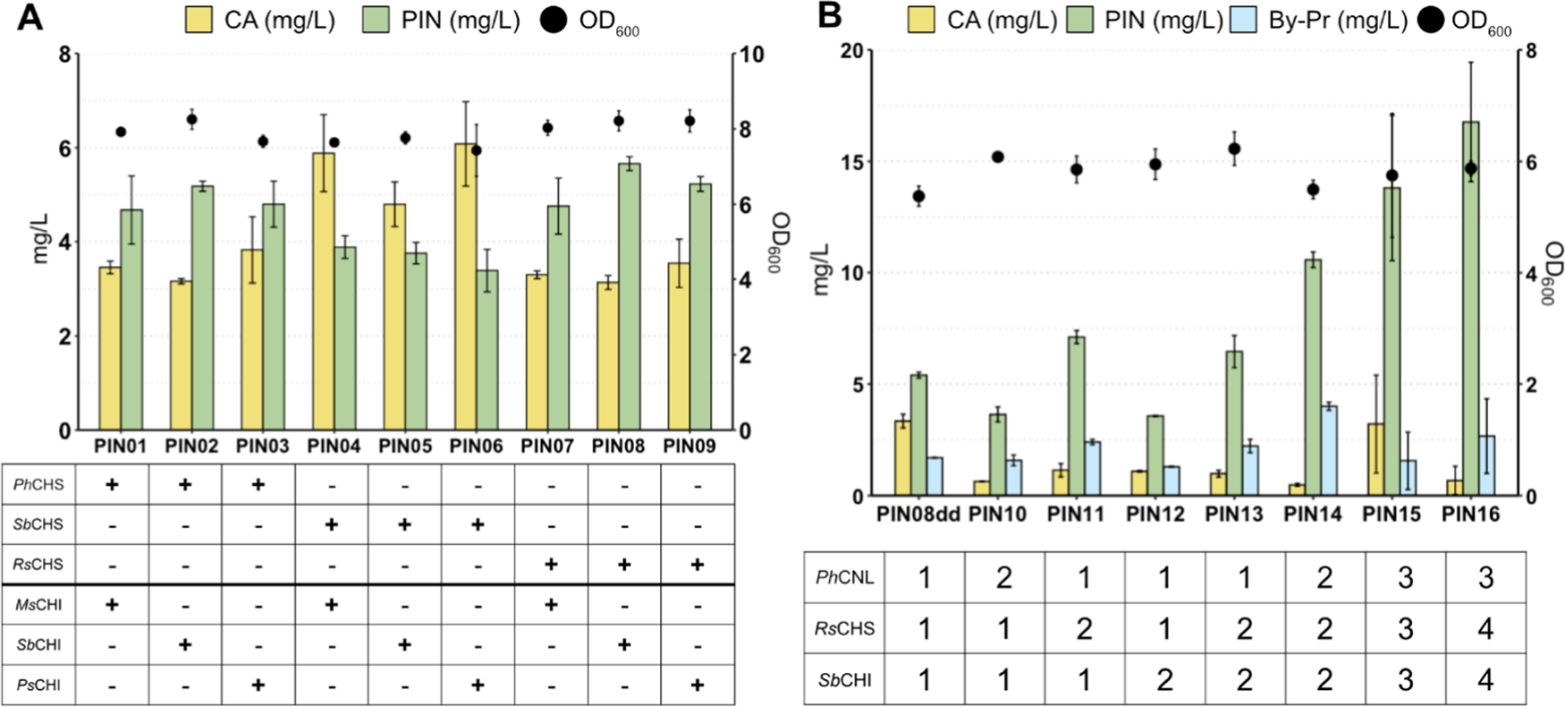
Optimizing the conversion of cinnamic acid to pinocembrin. (A) Pinocembrin and cinnamic acid production by engineered strains expressing the core cinnamic acid and pinocembrin synthetic genes. (B) Pinocembrin production following the gradual increase in gene copy numbers. The strains were cultivated in batch for 72 h at 30 °C using defined minimal medium supplemented with 30 g/L glucose. Data represent the mean of *n* = 3 independent biological samples, and error bars denote the standard deviation. CA, cinnamic acid; PIN, pinocembrin; By-Pr, byproduct (2′,4′,6′-trihydroxy dihydrochalcone); OD_600_, optical density; CNL, cinnamate-CoA ligase; CHS, chalcone synthase; CHI, chalcone isomerase; Ph, *Petunia hybrida*; Rs, *Rhododendron simsii*; Ms, *Medicago sativa*; Ps, *Paeonia suffruticosa*; Sb, *Scutellaria baicalensis*.

Pinocembrin biosynthesis was further investigated in strain CA02 because it exhibited higher CA production but lower growth inhibition compared to strain CA06. The following enzymes involved in pinocembrin biosynthesis were screened: CNL from *Petunia hybrida* (*Ph*CNL), CHS from *P. hybrida* (*Ph*CHS), CHS from *Scutellaria baicalensis* (*Sb*CHS), CHS from *Rhododendron simsii* (*Rs*CHS), CHI from *Medicago sativa* (*Ms*CHI), CHI from *S. baicalensis* (*Sb*CHI), and CHI from *Paeonia suffruticosa* (*Ps*CHI) (Figure 3A). All heterologous enzymes successfully converted CA to pinocembrin, indicating that they were functional in *S. cerevisiae*. However, the strains performed differently: the highest pinocembrin production was observed in strains expressing *Rs*CHS, whereas CHI homologues had only a minor impact, with *Sb*CHI (PIN08) yielding 5.8 mg/L pinocembrin (Figure 3A). Because our goal was to channel the CA flux toward downstream products and thus minimize CA accumulation, we selected strain PIN08 for further engineering steps.

To block any pathways competing with CA degradation, the genes *FDC1* and *PAD1* were deleted from strain PIN08, thus generating strain PIN08dd. Deletion of these genes, which encode for a ferulic acid decarboxylase and a phenylacrylic acid decarboxylase, respectively, has been shown to increase CA accumulation in *S. cerevisiae*.^35^ Surprisingly, their deletion had no effect on CA production in our strains (Figure S2). Sequencing revealed a stop codon in the middle of the genes’ open reading frame in our CENPK.113-7D background strain, explaining their nonfunctional status. Nevertheless, the newly generated PIN08dd strain was selected for further optimization.

Considering that the strain PIN08dd produced only 5.8 mg/L pinocembrin and still had some CA left, we tested if expression of the enzymes downstream of CA was limiting the production of pinocembrin. Various copies of *Ph*CNL, *Rs*CHS, and *Sb*CHI were introduced into the genome of PIN08dd (Figure 3B). When two copies of these genes were introduced, generating strains PIN10, PIN11, and PIN12, respectively, the limiting step in pinocembrin production was identified as the conversion of cinnamoyl-CoA to a trihydroxychalcone by CHS. Strain PIN11, which had two copies of CHS, could produce almost twice as much pinocembrin as strains PIN10 and PIN12. When three copies of CNL and four copies each of CHS and CHI were introduced, the resulting strain (PIN16) channeled almost all CA toward pinocembrin production, whose yield was now 17 mg/L. This represented a 3-fold improvement relative to the parental strain PIN08dd. However, pinocembrin was accompanied by the production of THDHC, an unwanted byproduct (Figure 3B) absent in CA-producing strains (Figure S3).

### Increased Availability of the Precursor Malonyl-CoA Improves Pinocembrin Production

The synthesis of one molecule of pinocembrin requires three molecules of malonyl-CoA. Malonyl-CoA availability represents a bottleneck in the production of flavonoids by *S. cerevisiae*.^6,36,37^ To overcome this limitation, we sought to increase the availability of malonyl-CoA using two different strategies. In the first one, two malonate assimilation genes from *Rhizobium trifolii* (*Rt*matC and *Rt*matB)^38^ were integrated into the genome of PIN16, resulting in strain PIN21. *Rt*matC is a carrier protein that transports malonate from the medium into the cell, whereas *Rt*matB is a malonate synthase that converts malonate to malonyl-CoA. In the second approach, a mutated acetyl-coenzyme A carboxylase, mAcc1** (Acc1^ser659ala,ser1157ala^), characterized by an enhanced activity,^39^ was integrated into the genome of PIN16, generating strain PIN33.

**Figure 4:**
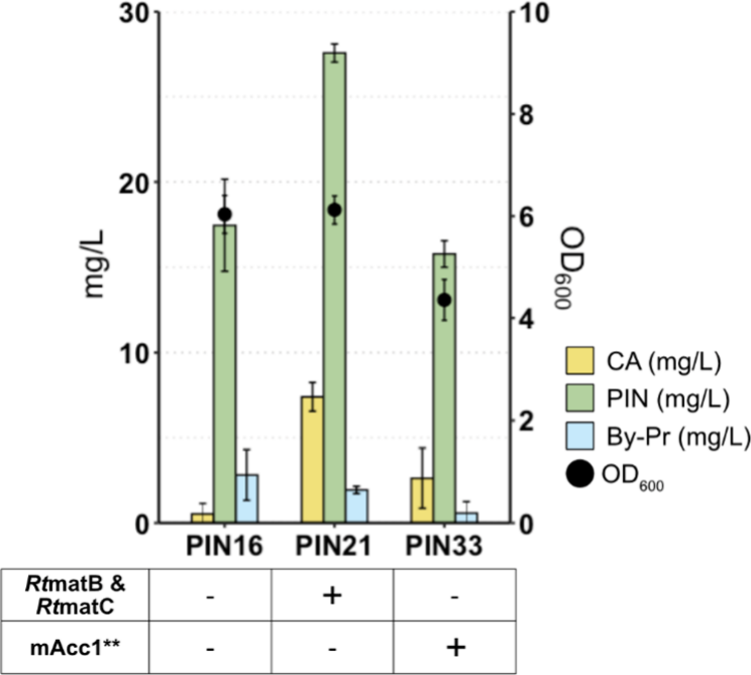
Increasing malonyl-CoA availability for pinocembrin production. Pinocembrin production following introduction of the genes RtmatB, RtmatC, and mAcc1**. The strains were cultivated in batch for 72 h at 30 °C using defined minimal medium supplemented with 30 g/L glucose. The medium for PIN21 was supplemented with 5 g/L malonate dibasic. Data represent the mean of *n* = 3 independent biological samples, and error bars denote the standard deviation. CA, cinnamic acid; PIN, pinocembrin; By-Pr, byproduct (2′,4′,6′-trihydroxy dihydrochalcone); OD_600_, optical density; Rt, *Rhizobium trifolii*; matC, malonate carrier protein; matB, malonate synthase protein; mAcc1*, mutated acetyl-coenzyme A carboxylase.

Strain PIN21 produced up to 27.56 mg/L pinocembrin (Figure 4), but it also accumulated 7.4 mg/L CA. After cultivating strain PIN21 for 72 h with supplemented malonate, the pH of the culture remained above 5, which contrasted with cultivation without malonate, whereby the pH was below 4.5 (Figure S4A). Given that the pKa of CA is 4.45, it is likely that at higher pH more CA was in a protonated form, which prevented its entry into the cell. Strain PIN33 displayed a similar production of pinocembrin as its PIN16 parent, but its growth was 30% lower (Figure 4). By normalizing to biomass, we calculated a 1.25-fold increase in pinocembrin production between strains PIN16 and PIN33 (Figure S5). To assess whether the intracellular concentration of malonyl-CoA increased in strains PIN21 and PIN33, we introduced a plasmid harboring a malonyl-CoA biosensor^40^ and detected the GFP signal using a flow cytometer. Fluorescence was 21 and 27.5% higher in strains PIN21 and PIN33 compared to strain PIN16, respectively (Figure S6). These results indicated that malonyl-CoA might be the limiting precursor in pinocembrin production. For further engineering purposes, we chose strain PIN33 as it did not require any additional external malonate and, thus, would not incur additional costs upon industrial scale-up. Moreover, strain PIN33 exhibited higher fluorescence and, therefore, malonyl-CoA concentration, than strain PIN21.

### Reduced Accumulation of the Undesired Byproduct Improves Pinocembrin Production

**Figure 5:**
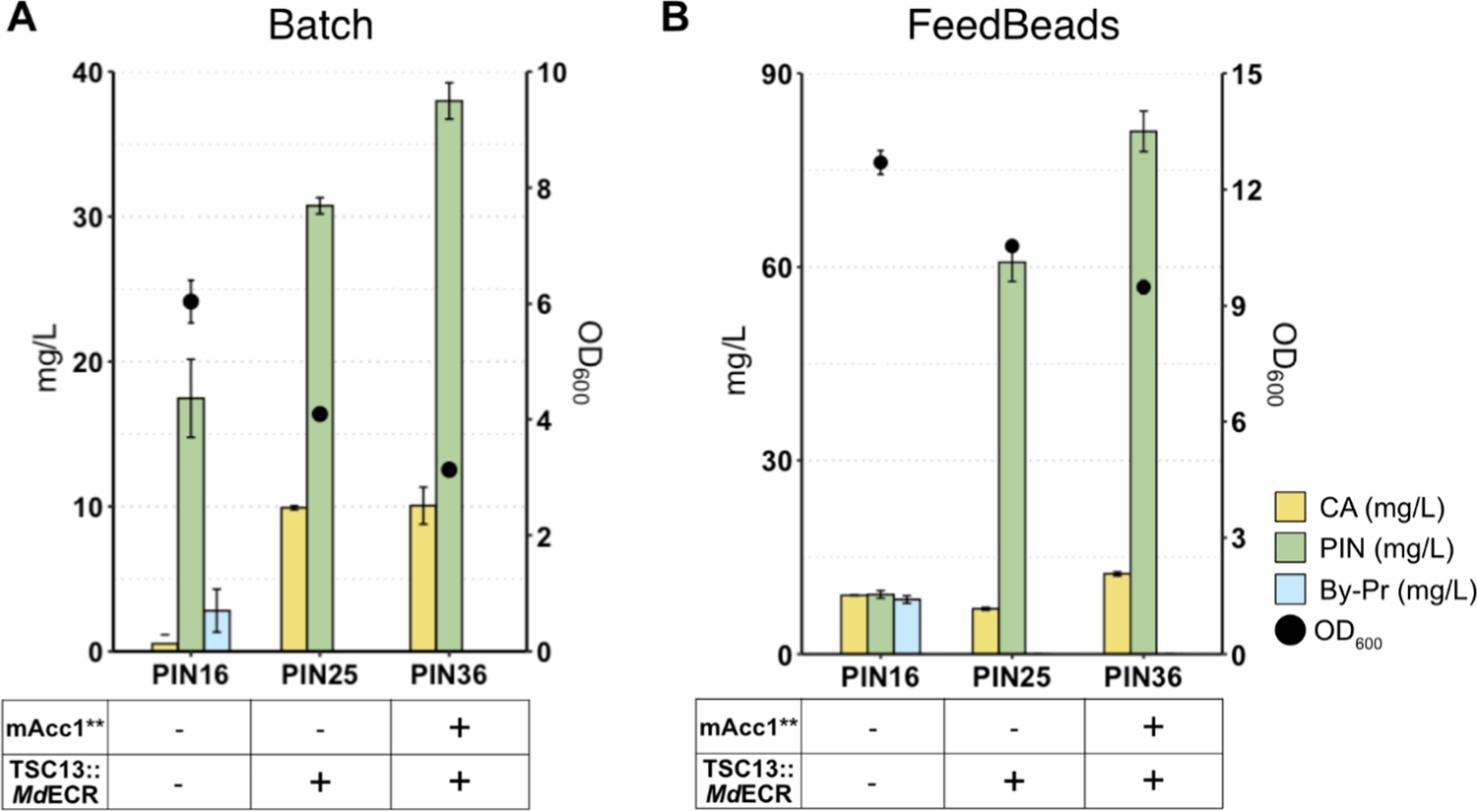
Reduction of undesired byproduct under batch and FeedBeads cultivation conditions. (A, B) Pinocembrin-producing strains, in which the native Tsc13 gene was replaced by MdECR. (A) Strains were cultivated in batch for 72 h at 30 °C using defined minimal medium supplemented with 30 g/L glucose. (B) Strains were cultivated in defined minimal medium supplemented with six tablets of FeedBeads as a sole carbon source for 96 h at 30 °C. Data represent the mean of *n* = 3 independent biological samples, and error bars denote the standard deviation. CA, cinnamic acid; PIN, pinocembrin; OD_600_, optical density; By-Pr, byproduct (2′,4′,6′-trihydroxy dihydrochalcone); Md, *Malus domestica*; TSC13, enoyl reductase; mAcc1*, mutated acetyl-coenzyme A carboxylase.

Although it has not been directly proven, cinnamoyl-CoA could serve as a substrate for the yeast’s native enoyl reductase Tsc13. Cinnamoyl-CoA shows high structural similarity to coumaroyl-CoA, and dihydrocinnamoyl-CoA can be condensed with 3 moles of malonyl-CoA by CHS (Figure 1). First, we confirmed that the byproduct formed in all our engineered pinocembrin-producing strains was consistent with the THDHC standard (Figure S3). To validate the biochemical conversions, a plant homologue, *Md*ECR from *Malus domestica*, was introduced to replace Tsc13 which catalyses the last step in each cycle of very-long-chain fatty acid elongation.^41^ This strategy has been reported to successfully prevent the formation of phloretic acid and phloretin in a naringenin-producing strain.^28^ Here, the *Md*ECR homologue replaced Tsc13 in strains PIN16 and PIN33, thereby generating strains PIN25 and PIN36, respectively. Strain PIN25 produced 1.6-fold more pinocembrin, but its growth was 33% lower. Instead, strain PIN36 increased pinocembrin production by 2.4-fold, while growth was reduced by 25%, along with an increased accumulation of CA compared to PIN33. Higher pinocembrin production in PIN36 compared to PIN25 could be ascribed to the overexpression of mAcc1**, which increased the intracellular malonyl-CoA pool. This indicates that malonyl-CoA remained a limiting factor even when carbon flux was diverted from THDHC to pinocembrin. Importantly, THDHC accumulation was reduced below detection in both PIN25 and PIN36 (Figure 5A).

As demonstrated before, *p*-HCA production could be significantly improved by enhancing the flux through the pentose phosphate pathway under glucose-limiting conditions.^31^ To determine if glucose limitation could stimulate pinocembrin production, we cultivated the strains under fed-batch-like conditions using FeedBeads as a slow-release form of glucose. Indeed, this cultivation mode boosted pinocembrin production to 60 mg/L (PIN25) and 80 mg/L (PIN36), corresponding to a 2-fold increase compared to batch conditions (Figure 5B).

### Extending the Pathway Downstream of Pinocembrin to Produce Chrysin

**Figure 6:**
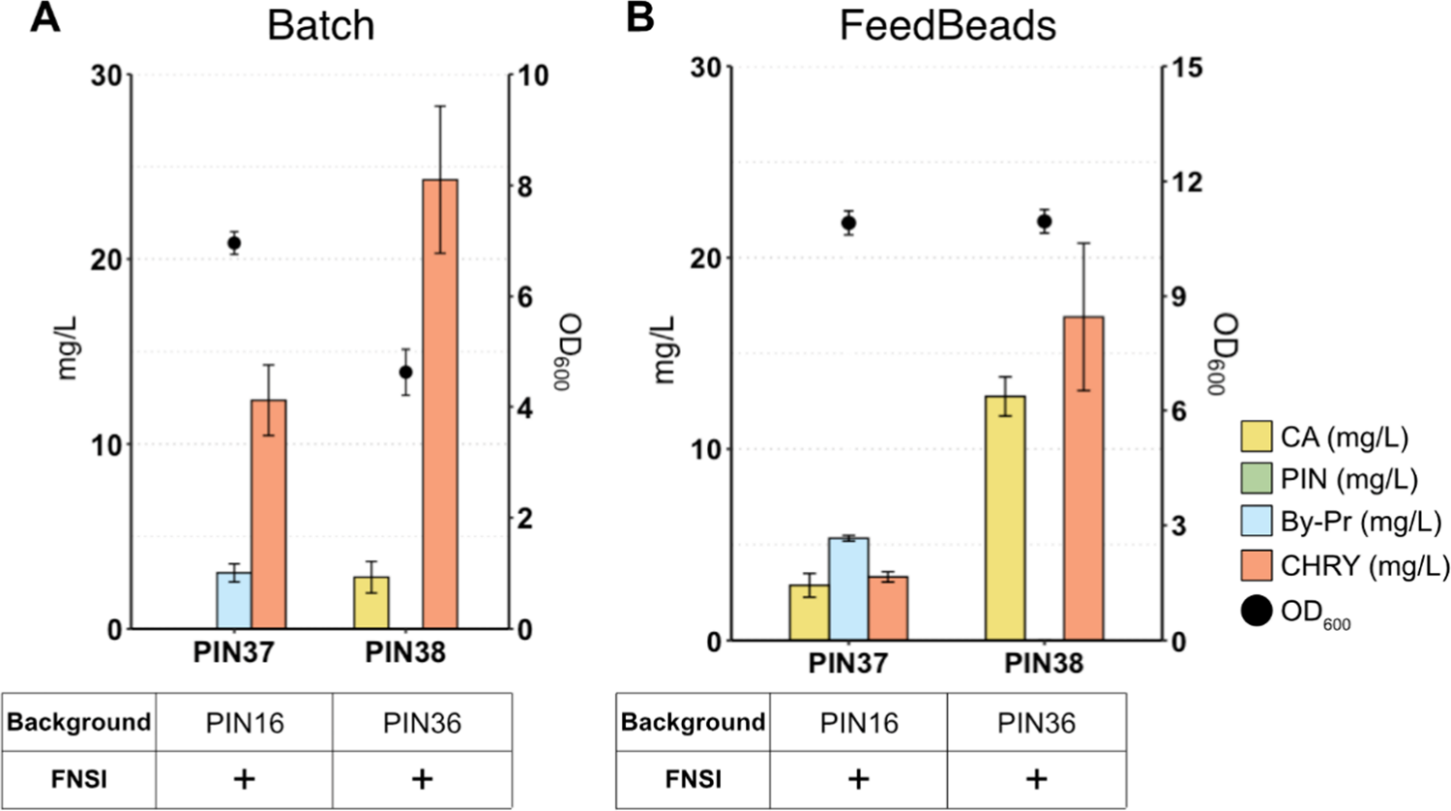
Chrysin production by engineered strains expressing genes needed to produce these flavones. (A) Strains were cultivated in batch for 72 h at 30 °C using defined minimal medium supplemented with 30 g/L glucose. (B) Strains were cultivated in defined minimal medium supplemented with six tables of FeedBeads as a sole carbon source for 96 h at 30 °C. Data represent the mean of *n* = 3 independent biological samples, and error bars denote the standard deviation. CA, cinnamic acid; PIN, pinocembrin; OD_600_, optical density; By-Pr, byproduct (2′,4′,6′-trihydroxy dihydrochalcone); Chry, chrysin; FNSI, flavone synthase I.

Pinocembrin producer strains can be used as a platform to generate other downstream flavones. As a proof of concept, we used strains PIN16 and PIN36 to produce chrysin (Figure 6). To this end, two enzymes were tested: flavone synthase I (FNSI) from *Petroselinum crispum*, which is found in soluble form in the cytosol,^42^ and flavone synthase II (FNSII) from *S. baicalensis*, which is a membrane-bound cytochrome P450-dependent monooxygenase.^43^ After genomic integration of both genes and chrysin quantification, FNSI emerged as the one allowing for higher chrysin output (Figure S7), almost complete conversion of pinocembrin, and very low levels of the CA. The resulting strain derived from PIN16 (PIN37) transformed 16 mg/L pinocembrin to 12 mg/L chrysin, whereas the strain derived from PIN36 (PIN38) could successfully convert 40 mg/L pinocembrin to 25 mg/L chrysin (Figure 6A). Unexpectedly, cultivation of PIN37 and PIN38 under glucose-limiting conditions with FeedBeads (Figure 6B) yielded less chrysin than batch cultivation despite a significant increase in biomass. Also, a significant increase in CA was detected upon FeedBeads cultivation. Thus, additional optimization is required to increase chrysin production.

## Discussion

At present, most research is focused on producing flavonoids derived from *p*-HCA such as naringenin.^8,10^ However, derivatives of CA, such as pinocembrin and chrysin, have equally interesting pharmaceutical and nutraceutical properties and are currently produced at a very low level in *S. cerevisiae*.^17,19,20,44^ Our results identified CA as the main bottleneck in the production of pinocembrin and its derivatives by *S. cerevisiae*. Indeed, the cells produced 10 times less CA than *p*-HCA, which is obtained from CA through hydroxylation (Figure 2A). A similar observation was reported by Li et al., when attempting to produce baicalein (23.6 mg/L), a derivative of CA, and scutellarein (106.5 mg/L), a derivative of *p*-HCA, in *E. coli*.^45^ This disparity can be explained by CA being toxic to cells and impairing growth.^29,34^ The same reasoning has been extrapolated to the production of pinocembrin derivatives,^24,25,46^ although no experimental or toxicological data support this hypothesis. In this study, cell growth was inhibited by a significantly lower concentration of CA (Figure 2C) than reported under pH-buffered conditions in a shake flask^29^ or bioreactor,^34^ suggesting an effect of pH on CA toxicity.

The molecular mechanism through which CA impairs cell fitness remains unknown. A common strategy to alleviate CA accumulation is to channel CA toward less toxic downstream products, such as cinnamyl alcohol and hydrocinnamyl alcohol in *S. cerevisiae* ^29,30^ or pinocembrin in *E. coli*.^21,47^ To produce pinocembrin from CA in *S. cerevisiae*, several enzymes from different hosts were screened (Figure 3A), echoing earlier attempts using enzymes from *P. crispum*, *P. hybrida*,^24^*S. baicalensis*, and *Erigeron breviscapus*.^25,46^ Here, we generated nine strains that combined CNL, CHS, and CHI from different organisms, of which *R. simsii* and *S. baicalensis* exhibited the strongest activity. However, because single-copy integration of these enzymes is usually not enough to reach high titers of flavonoids, multiple copies are usually integrated. This is the case of naringenin titers in *S. cerevisiae*.^11^ In the present study, expression of CNL, CHS, and CHI at a 3:4:4 ratio increased 3-fold the titer of pinocembrin, while significantly decreasing the accumulation of CA (Figure 3B).

Availability of malonyl-CoA is another limiting factor for high-level production of pinocembrin. The introduction of a bacterial malonate assimilation pathway or a boost in acetyl-CoA carboxylase activity improved the supply of malonyl-CoA (Figure S6) and pinocembrin production (Figure 4). This, however, was accompanied by the accumulation of more extracellular CA (Figure S4). The same effect was observed in strain PIN36, which did not contain a malonate assimilation pathway and could exclude any metabolic effect of malonate. This phenomenon may be related to a higher buffering capacity of medium containing dibasic malonate, as suggested by the final pH being higher than without malonate (Figure S4). At low pH, uncharged weak acids can diffuse into the cell, where they encounter a more neutral pH and become dissociated, thus leading to intracellular acidification and the inhibition of metabolic processes.^48^ In contrast, at higher pH, weak acids accumulate outside the cell, which may lower the toxicity of CA, as indicated by a comparison of CA tolerance at pH 7.1 vs pH 4.0.^49^ Controlling the pH of the medium could improve pinocembrin titers, but the extracellular accumulation of CA still represents a waste of carbon for *de novo* synthesis of pinocembrin and its derivatives.

Another limitation of this pathway is represented by the production of THDHC as a side product. We show that inhibition of this reaction diverted the carbon flux toward pinocembrin, whose concentration increased 1.6-fold (Figure 5). Even though THDHC has been detected before,^27^ blocking its production to improve the biosynthesis of pinocembrin in *S. cerevisiae* has not been attempted.^24,25^

The production of pinocembrin derivatives has been demonstrated in *E. coli* and *S. cerevisiae*. In bacteria, this is especially complicated because it requires the optimization of P450 enzymes. By truncating the N-terminus of flavonoid 6-hydroxylase from *S. baicalensis* and cytochrome P450 reductase from *A. thaliana*, a titer of 8.5 mg/L baicalein was achieved in *E. coli*.^45^ Most recently, the same group developed a self-assembly enzyme reactor^50^ and, together with transcriptome-assisted modularization, augmented the titer to 367.8 mg/L baicalein under continuous fed-batch fermentation in a bioreactor.^51^ Instead, engineered *S. cerevisiae* strains have achieved at the most 7.25 mg/L chrysin, 4.69 mg/L baicalein, and 5.53 mg/L baicalin.^25^ Therefore, successful, large-scale production of pinocembrin derivatives in this host remains a challenge.

In conclusion, in this study, we first identified the limitations hampering *de novo* pinocembrin biosynthesis from glucose. Then, by addressing these bottlenecks, we improved pinocembrin production to 80 mg/L in shake flasks. Even though the titer is still lower than with other hosts, our study nevertheless demonstrates the feasibility of increasing pinocembrin production in *S. cerevisiae*. The engineered strains may, therefore, serve as a platform for the characterization of novel plant-derived enzymes from which to produce other promising pinocembrin derivatives.

## Methods

### Strains and Reagents

All strains and plasmids used in this study are listed in Table S1. All chemicals including analytical standards were purchased from Sigma-Aldrich. For PCRs, high-fidelity Phusion DNA polymerase was purchased from New England Biolabs, while PrimeStar DNA polymerase and Sapphire AmpFast PCR Master Mix were purchased from TaKaRa Bio. To clean up PCR DNA products and for plasmid extractions, kits were purchased from Thermo Fisher Scientific. All oligonucleotides used in this study were purchased from Eurofins and are listed in Table S2. All codon-optimized heterologous genes were synthesized at Genscript and are listed in Table S3.

### Strain Engineering

All strains published in this work are derivatives of IMX581 (MATa ura3-52 can1Δ::cas9-natNT2 TRP1 LEU2 HIS3) and were engineered with CRISPR-Cas9 technology.^33^ All native promoters and terminators were amplified using IMX581 genomic DNA as template. For each cassette, an upstream region from the guide RNA (gRNA) cutting site (∼500 bp), a promoter, a gene, a terminator, and a downstream region from the gRNA cutting site (∼500 bp) were first amplified by PCR (using high-fidelity Phusion DNA polymerase). Second, all linear DNA fragments were fused together by overlapping extension PCR (using PrimeSTAR HS polymerase).^52^ All integration cassettes and oligonucleotides used in these steps are listed in Tables S2 and S4. The integration sites used in this study are listed in Table S4; they have been proven to be stable and enable strong expression of heterologous genes.^53^ The gRNAs used in this study are listed in Table S5.

### Strain Cultivation

To prepare *S. cerevisiae* competent cells, strains were cultivated in YPD medium consisting of 10 g/L yeast extract (Merck Millipore), 20 g/L peptone (Difco), and 20 g/L glucose (Merck Millipore). To select transformants containing URA3 marker-based plasmids, synthetic complete medium without uracil was used. This medium consisted of 6.7 g/L yeast nitrogen base without amino acids (Formedium), 0.77 g/L CSM without uracil (Formedium), 20 g/L glucose (Merck Millipore), and 20 g/L agar (Merck Millipore). To lose the URA3 marker plasmid, yeast transformants were selected on synthetic complete medium with 5-fluoroorotic acid, which contained 6.7 g/L yeast nitrogen base, 0.77 g/L CSM, and 0.8 g/L 5-FOA.

To produce cinnamic acid, pinocembrin, and chrysin, batch fermentations were performed in 100 mL shake flasks using minimal medium containing 7.5 g/L (NH_4_)_2_SO_4_, 14.4 g/L KH_2_PO_4_, 0.5 g/L MgSO_4_·7H_2_O, 30 g/L glucose, 2 mL/L trace metals (3.0 g/L FeSO_4_·7H_2_O, 4.5 g/L ZnSO_4_·7H_2_O, 4.5 g/L CaCl_2_·2H_2_O, 0.84 g/L MnCl_2_·2H_2_O, 0.3 g/L CoCl_2_·6H_2_O, 0.3 g/L CuSO_4_·5H_2_O, 0.4 g/L Na_2_MoO_4_·2H_2_O, 1.0 g/L H_3_BO_3_, 0.1 g/L KI, and 19.0 g/L Na_2_EDTA·2H_2_O), and 1 mL/L vitamin solutions (0.05 g/L d-biotin, 1.0 g/L d-pantothenic acid hemicalcium salt, 1.0 g/L thiamin–HCl, 1.0 g/L pyridoxin–HCl, 1.0 g/L nicotinic acid, 0.2 g/L 4-aminobenzoic acid, and 25.0 g/L myo-inositol) plus 120 mg/L uracil if needed. When shake flasks were used to mimic fed-batch conditions, 6 FeedBeads tablets (SMFB08001; Kuhner Shaker, Basel, Switzerland) corresponding to 30 g/L glucose, were used as the sole carbon source, and cultivations were run for 96 h at 30 °C with 220 rpm agitation.

For each round of experiments with engineered strains, three biological replicates were inoculated in cultivation tubes containing 2 mL minimal medium and were grown at 30 °C under 220 rpm agitation for 24 h. Afterward, the precultures were inoculated to an initial OD_600_ of 0.02 in 20 mL minimal medium inside 100 mL unbaffled shake flasks. When needed, 5 g/L dibasic sodium malonate (Sigma-Aldrich) was supplemented. The cells were cultivated at 30 °C and 220 rpm for 72 h.

### Metabolite Extraction and Quantification

To extract the flavonoids of interest (CA, pinocembrin, and chrysin), 0.5 mL of cell culture from the shake flask fermentation was mixed with absolute ethanol (100% v/v), vortexed for 5 min, and centrifuged at 15,000 rpm and 4 °C. The supernatants were used to quantify the products on a high-performance liquid chromatographer (Thermo Fisher Scientific) coupled to a photodiode array detector and equipped with a Discovery HS F5 150 mm × 46 mm column (particle size 5 μm; Sigma-Aldrich). Solvent A was 10 mM ammonium formate (pH 3, adjusted with formic acid), and solvent B was acetonitrile. The eluent flow rate was 1.5 mL/min. The elution gradient started with 5% solvent B (0–0.5 min), followed by a linear increase from 5 to 60% solvent B (0.5–20.5 min), another linear increase from 60 to 100% solvent B (20.5–21.5 min), maintenance at 100% solvent B for 1 min (21.5–22.5 min), a linear decrease from 100 to 5% solvent B (22.5–23.5 min), and maintenance at 5% solvent B for 0.5 min (23.5–24 min). CA, pinocembrin, THDHC, and chrysin were detected at 289 nm, whereby they exhibited a retention time of 12.5, 18.7, 18.9, and 18.2 min, respectively. Their concentration was calculated based on standard curves.

## Supporting Information Available

HPLC chromatogram of the *p*-coumaric acid producer strain after 72 h cultivation; chromatogram of the standard mix used in this study; chromatogram of the standard that includes *p*-coumaric acid; metabolite concentrations in strains where the genes to produce styrene from cinnamic acid are deleted; chromatograms comparing the production profile of the strains CA06 and PIN16 with the standards; effect of malonate dibasic supplementation when cultivating PIN21 and PIN36 strain; normalized production of flavonoids to the growth of strains with an increased pool of precursor malonyl-CoA; evaluating malonyl-CoA supply using two different strategies using malonyl-CoA sensor; metabolite concentrations of chrysin production strains expressing two different flavone synthases; and strains, primers, codon-optimized heterologous gene sequences, expression cassettes and gRNAs used in this study (PDF)

## Abbreviations

CA: cinnamic acid
*p*-HCA: *p*-coumaric acid
By-Pr: byproduct
PIN: pinocembrin
Chry: chrysin
THDHC: 2′,4′,6′-trihydroxy dihydrochalcone
CNL: cinnamate-CoA ligase
CHS: chalcone synthase
CHI: chalcone isomerase
