# Supplementary material for: Optimization of Pinocembrin Biosynthesis in Saccharomyces cerevisiae
Source: ACS Synth Biol. 2022 Dec 19;12(1):144–52. doi: 10.1021/acssynbio.2c00425 (PMC9872169; doi:10.1021/acssynbio.2c00425)
Supplement: Supplementary file 1 — sb2c00425_si_001.pdf [file sb2c00425_si_001.pdf]

Optimisation of pinocembrin biosynthesis in *Saccharomyces cerevisiae*

Marta Tous Mohedano[1#], Jiwei Mao[1#], Yun Chen[1*]

[1]Department of Biology and Biological Engineering, Chalmers University of Technology, Göteborg, SE41296, Sweden

[#] These authors contributed equally

*Corresponding author
Email: yunc@chalmers.se

Figure S1

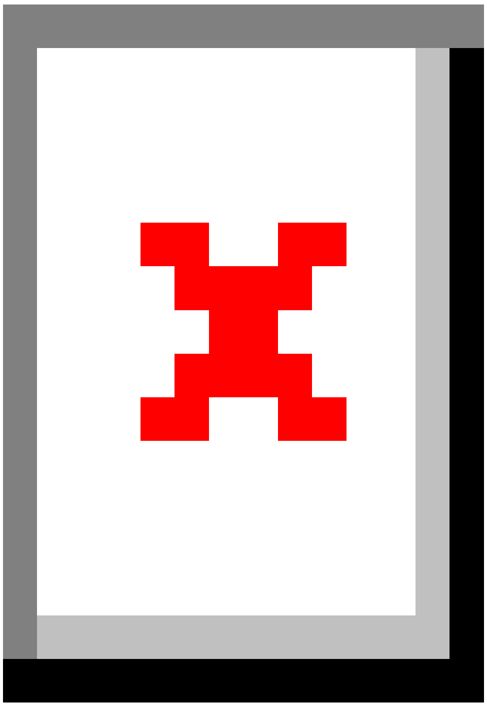

Figure S1 ***. (A) Chromatogram of the strain QL12 after 72h cultivation, (B) chromatogram of the standard mix that includes cinnamic acid (elutes at 12.44 min), pinocembrin (elutes at 17.55 min) and by- product (elutes at 17.85 min), (C) chromatogram of the standard that includes p-coumaric acid (elutes at 8.86 min).*** *In the strain QL12 the p- coumaric acid peak elutes at 8.86 minutes and there is no other compound detected in this sample.*

Figure S2.

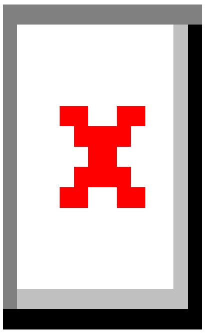

Figure S2. ***Deletion of genes to produce styrene from cinnamic acid****. The strains were cultivated in batch defined minimal media for 72 h at 30°C with 30 g/L glucose. All data represent the mean of n=3 biological independent samples and error bars show the standard deviation. CA, cinnamic acid; PIN, pinocembrin; AAA^{E}, overexpression of Aro7^{G141S}, Aro4^{K229L}, ARO1/2/3, PHA2, EcAroL from E. coli; FDC1, ferulic acid decarboxylase and PAD1, phenylacrylic acid decarboxylase.*

Figure S3

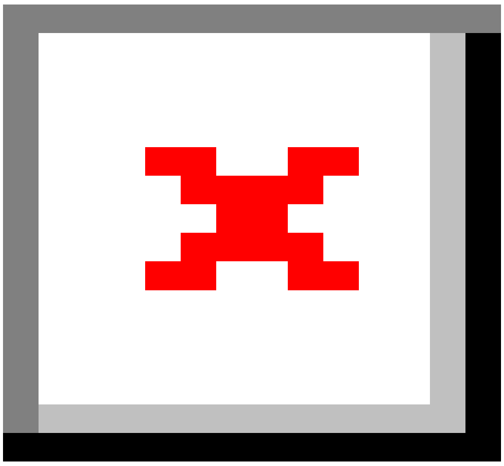

Figure S3 ***Comparison of the chromatograms of the standard mix showing CA, PIN, and by-product (A), chromatogram of strain CA06 after 72h cultivation(B) chromatogram of strain PIN16 after 72h cultivation (C).*** *The chromatogram of the standard mix (A) shows the cinnamic acid peak that elutes at 12.44 min, the pinocembrin peak that elutes at 17.55 minutes and the by-product peak that elutes at 17.85 minutes. (B) shows that the strain CA06 after 72h cultivation presents only the CA peak that elutes at 12.55 min. (C) shows that the strain PIN16 after 72h cultivation shows a Pinocembrin peak that elutes at 17.55 min and a by-product peak that elutes at 17.85 min.*

Figure S4.

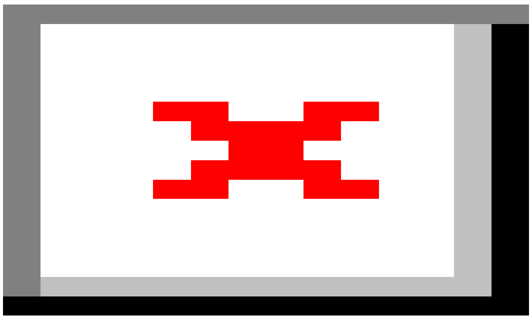

Figure S4. ***Effect of malonate dibasic supplementation when cultivating PIN21(A) and PIN36(B) strain****. The strains were cultivated in batch defined minimal media for 72 h at 30°C with 30 g/L glucose. The media of the strains PIN21_M and PIN36_M was supplemented with 5 g/L malonate dibasic. The samples for flavonoid quantification, pH and OD_{600} measurements were done after 72h of cultivation. CA, cinnamic acid; PIN, pinocembrin; By-Pr, by-product (2',4',6'-trihydroxy dihydrochalcone); OD_{600}, optical density.*

Figure S5.

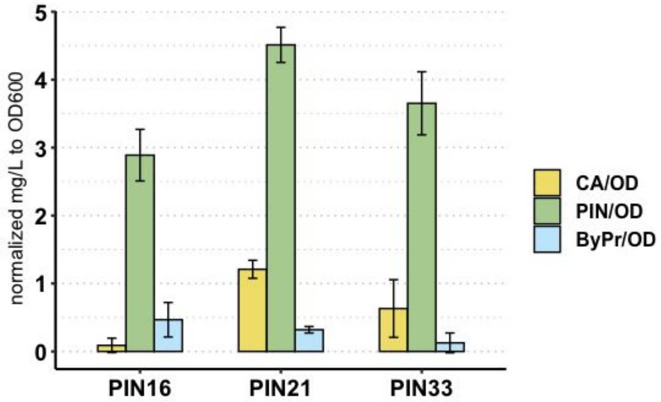

CNL:CHS:CHI	3:4:4	3:4:4	3:4:4
***Rt*matB & *Rt*matC**	-	+	-
**mAcc1****	-	-	+

Figure S5. ***Normalized production of flavonoids to the growth on strains with an increased pool of precursor malonyl-CoA****. Pinocembrin production normalized to OD_{600} when the genes RtmatB, RtmatC and mAcc1** were introduced. The strains were cultivated in batch defined minimal media for 72 h at 30°C with 30 g/L glucose. The media of PIN21 was supplemented with 5 g/L malonate dibasic. All data represent the mean of n=3 biological independent samples and error bars show the standard deviation. The first raw indicates the ratio of the enzymes CNL:CHS:CHI in the strains. CA, cinnamic acid; PIN, pinocembrin; ByPr, by-product (2',4',6'-trihydroxy dihydrochalcone); OD_{600}, optical density; CNL, cinnamate-CoA ligase; CHS, chalcone synthase; CHI, chalcone isomerase; Rt, Rhizobium trifolii; matC, malonate carrier protein; matB, malonate synthase protein; mAcc1*, mutated acetyl-coenzyme A carboxylase*

Figure S6.

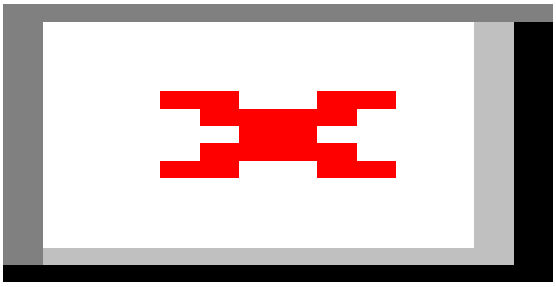

Figure S6. Evaluating malonyl-CoA supply of two different strategies by using malonyl-CoA sensor. (A) Flow cytometry measurement of different strains carrying the malonyl-CoA biosensor plasmid. (B) Quantitative GFP intensity of malonyl-CoA sensor in different strains. All strains were cultured in defined minimal medium with 30 g/L glucose as the sole carbon source, and with 2 g/L sodium malonate dibasic supplementation when required. Fluorescence was measured at 12h after inoculation. All data represent the mean of n= 3 biologically independent samples and error bars show standard deviation.

Figure S7.

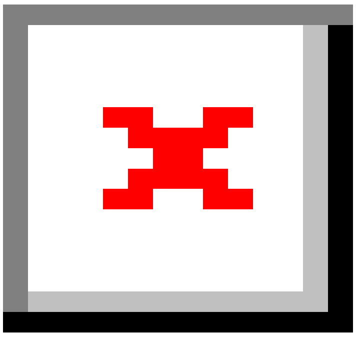

Figure S7. ***Chrysin production strains expressing 2 different flavone synthases.*** *The strains were cultivated in batch defined minimal media for 72 h at 30°C with 30 g/L glucose. All data for strain PIN37 represent the mean of at least n=3 biological independent samples and error bars show the standard deviation. For strain CHRY2 only 2 clones produced chrysin as a final product therefore the data for this strain represents the mean of 2 biological independent samples and the error bars show the standard deviation. FNSI, flavone synthase I; FNSII, flavone synthase II; CA, cinnamic acid; PIN, pinocembrin; Chry, chrysin;* OD_{600}*, optical density; By-Pr, by-product (2',4',6-'trihydroxy dihydrochalcone).*

Supplementary Table 1. S. cerevisiae strains used in this study

Strain ID	Genotype	Parental strain	Reference
**IMX581**	MATa ura3-52 can1Δ :: cas9-natNT2 TRP1 LEU2 HIS3		1
**QL01**	MATa ura3-52 can1Δ::cas9-natNT2 TRP1 LEU2 HIS3 XII-2::(GPM1p-AtPAL2-FBA1t)+(TDH3p-AtC4H-CYC1t)+(tHXT7p-AtATR2-pYX212t)+(PGK1p-CYB5-ADH1t)	IMX581	2
**QL12**	MATa ura3-52 can1Δ::cas9-natNT2 TRP1 LEU2 HIS3 X-3::(TPI1p-EcaroL-pYX212t)+(ADH1t-ARO7 G141S-TEF1p)+(PGK1p-ARO4 K229L-CYC1t) X-4::(CYC1t-ARO1-TPI1p)+(TDH3p-ARO2-ADH1t)+(TDH2t-ARO3-TEF1p) X-2::(GPM1p-PHA2-CYC1t), XII-2::(GPM1p-AtPAL2-FBA1t)+(TDH3p-AtC4H-CYC1t)+(tHXT7p-AtATR2-pYX212t)+(PGK1p-CYB5-ADH1t)		2
**CA01**	MATa ura3-52 can1Δ::cas9-natNT2 TRP1 LEU2 HIS3 XII-2::(GPM1p-AtPAL2-FBA1t)	IMX581	This study
**CA02**	MATa ura3-52 can1Δ::cas9-natNT2 TRP1 LEU2 HIS3 X-3::(TPI1p-EcaroL-pYX212t)+(ADH1t-ARO7 G141S-TEF1p)+(PGK1p-ARO4 K229L-CYC1t) X-4::(CYC1t-ARO1-TPI1p)+(TDH3p-ARO2-ADH1t)+(TDH2t-ARO3-TEF1p) X-2::(GPM1p-PHA2-CYC1t) XII-5::(GPM1p-AtPAL2-FBA1t)	CA01	This study
**CA03**	MATa ura3-52 can1Δ::cas9-natNT2 TRP1 LEU2 HIS3 X-3::(TPI1p-EcaroL-pYX212t)+(ADH1t-ARO7 G141S-TEF1p)+(PGK1p-ARO4 K229L-CYC1t) X-4::(CYC1t-ARO1-TPI1p)+(TDH3p-ARO2-ADH1t)+(TDH2t-ARO3-TEF1p) X-2::(GPM1p-PHA2-CYC1t) XII-5::(GPM1p-RtPAL2-FBA1t)	CA01	This study
**CA04**	MATa ura3-52 can1Δ::cas9-natNT2 TRP1 LEU2 HIS3 X-3::(TPI1p-EcaroL-pYX212t)+(ADH1t-ARO7 G141S-TEF1p)+(PGK1p-ARO4 K229L-CYC1t) X-4::(CYC1t-ARO1-TPI1p)+(TDH3p-ARO2-ADH1t)+(TDH2t-ARO3-TEF1p) X-2::(GPM1p-PHA2-CYC1t), XII-2::(GPM1p-AtPAL2-FBA1t), XII-5::(GPM1p-RtPAL2-FBA1t)	CA02	This study
**CA05**	MATa ura3-52 can1Δ::cas9-natNT2 TRP1 LEU2 HIS3 X-3::(TPI1p-EcaroL-pYX212t)+(ADH1t-ARO7 G141S-TEF1p)+(PGK1p-ARO4 K229L-CYC1t) X-4::(CYC1t-ARO1-TPI1p)+(TDH3p-ARO2-ADH1t)+(TDH2t-ARO3-TEF1p) X-2::(GPM1p-PHA2-CYC1t), XII-2::(GPM1p-AtPAL2-FBA1t), XII-5::(GPM1p-AtPAL2-FBA1t)	CA02	This study
**CA06**	MATa ura3-52 can1Δ::cas9-natNT2 TRP1 LEU2 HIS3 X-3::(TPI1p-EcaroL-pYX212t)+(ADH1t-ARO7 G141S-TEF1p)+(PGK1p-ARO4 K229L-CYC1t) X-4::(CYC1t-ARO1-TPI1p)+(TDH3p-ARO2-ADH1t)+(TDH2t-ARO3-TEF1p) X-2::(GPM1p-PHA2-CYC1t), XII-2::(GPM1p-AtPAL2-FBA1t), XII-5::(GPM1p-AtPAL2-FBA1t)+(CYC1t-AtPAL2-TP1p)	CA05	This study
**PIN01**	MATa ura3-52 can1Δ::cas9-natNT2 TRP1 LEU2 HIS3 X-3::(TPI1p-EcaroL-pYX212t)+(ADH1t-ARO7 G141S-TEF1p)+(PGK1p-ARO4 K229L-CYC1t) X-4::(CYC1t-ARO1-TPI1p)+(TDH3p-ARO2-ADH1t)+(TDH2t-ARO3-TEF1p) X-2::(GPM1p-PHA2-CYC1t) XII-5::(GPM1p-AtPAL2-FBA1t) XII-4::(CYC1t-PhCNL-TEF1p)+(PGK1p-PhCHS-ADH1t)+(TDH2t-MsCHI2-TDH3p)	CA02	This study
**PIN02**	MATa ura3-52 can1Δ::cas9-natNT2 TRP1 LEU2 HIS3 X-3::(TPI1p-EcaroL-pYX212t)+(ADH1t-ARO7 G141S-TEF1p)+(PGK1p-ARO4 K229L-CYC1t) X-4::(CYC1t-ARO1-TPI1p)+(TDH3p-ARO2-ADH1t)+(TDH2t-ARO3-TEF1p) X-2::(GPM1p-PHA2-CYC1t) XII-5::(GPM1p-AtPAL2-FBA1t) XII-4::(CYC1t-PhCNL-TEF1p)+(PGK1p-PhCHS-ADH1t)+(TDH2t-SbCHI2-TDH3p)	CA02	This study

**PIN03**	MATa ura3-52 can1Δ::cas9-natNT2 TRP1 LEU2 HIS3 X-3::(TPI1p-EcaroL-pYX212t)+(ADH1t-ARO7 G141S-TEF1p)+(PGK1p-ARO4 K229L-CYC1t) X-4::(CYC1t-ARO1-TPI1p)+(TDH3p-ARO2-ADH1t)+(TDH2t-ARO3-TEF1p) X-2::(GPM1p-PHA2-CYC1t) XII-5::(GPM1p-AtPAL2-FBA1t) XII-4::(CYC1t-PhCNL-TEF1p)+(PGK1p-PhCHS-ADH1t)+(TDH2t-PsCHI2-TDH3p)	CA02	This study
**PIN04**	MATa ura3-52 can1Δ::cas9-natNT2 TRP1 LEU2 HIS3 X-3::(TPI1p-EcaroL-pYX212t)+(ADH1t-ARO7 G141S-TEF1p)+(PGK1p-ARO4 K229L-CYC1t) X-4::(CYC1t-ARO1-TPI1p)+(TDH3p-ARO2-ADH1t)+(TDH2t-ARO3-TEF1p) X-2::(GPM1p-PHA2-CYC1t) XII-5::(GPM1p-AtPAL2-FBA1t) XII-4::(CYC1t-PhCNL-TEF1p)+(PGK1p-SbCHS-ADH1t)+(TDH2t-MsCHI2-TDH3p)	CA02	This study
**PIN05**	MATa ura3-52 can1Δ::cas9-natNT2 TRP1 LEU2 HIS3 X-3::(TPI1p-EcaroL-pYX212t)+(ADH1t-ARO7 G141S-TEF1p)+(PGK1p-ARO4 K229L-CYC1t) X-4::(CYC1t-ARO1-TPI1p)+(TDH3p-ARO2-ADH1t)+(TDH2t-ARO3-TEF1p) X-2::(GPM1p-PHA2-CYC1t) XII-5::(GPM1p-AtPAL2-FBA1t) XII-4::(CYC1t-PhCNL-TEF1p)+(PGK1p-SbCHS-ADH1t)+(TDH2t-SbCHI2-TDH3p)	CA02	This study
**PIN06**	MATa ura3-52 can1Δ::cas9-natNT2 TRP1 LEU2 HIS3 X-3::(TPI1p-EcaroL-pYX212t)+(ADH1t-ARO7 G141S-TEF1p)+(PGK1p-ARO4 K229L-CYC1t) X-4::(CYC1t-ARO1-TPI1p)+(TDH3p-ARO2-ADH1t)+(TDH2t-ARO3-TEF1p) X-2::(GPM1p-PHA2-CYC1t) XII-5::(GPM1p-AtPAL2-FBA1t) XII-4::(CYC1t-PhCNL-TEF1p)+(PGK1p-SbCHS-ADH1t)+(TDH2t-PsCHI2-TDH3p)	CA02	This study
**PIN07**	MATa ura3-52 can1Δ::cas9-natNT2 TRP1 LEU2 HIS3 X-3::(TPI1p-EcaroL-pYX212t)+(ADH1t-ARO7 G141S-TEF1p)+(PGK1p-ARO4 K229L-CYC1t) X-4::(CYC1t-ARO1-TPI1p)+(TDH3p-ARO2-ADH1t)+(TDH2t-ARO3-TEF1p) X-2::(GPM1p-PHA2-CYC1t) XII-5::(GPM1p-AtPAL2-FBA1t) XII-4::(CYC1t-PhCNL-TEF1p)+(PGK1p-RsCHS-ADH1t)+(TDH2t-SbCHI2-TDH3p)	CA02	This study
**PIN08**	MATa ura3-52 can1Δ::cas9-natNT2 TRP1 LEU2 HIS3 X-3::(TPI1p-EcaroL-pYX212t)+(ADH1t-ARO7 G141S-TEF1p)+(PGK1p-ARO4 K229L-CYC1t) X-4::(CYC1t-ARO1-TPI1p)+(TDH3p-ARO2-ADH1t)+(TDH2t-ARO3-TEF1p) X-2::(GPM1p-PHA2-CYC1t) XII-5::(GPM1p-AtPAL2-FBA1t) XII-4::(CYC1t-PhCNL-TEF1p)+(PGK1p-RsCHS-ADH1t)+(TDH2t-SbCHI2-TDH3p)	CA02	This study
**PIN09**	MATa ura3-52 can1Δ::cas9-natNT2 TRP1 LEU2 HIS3 X-3::(TPI1p-EcaroL-pYX212t)+(ADH1t-ARO7 G141S-TEF1p)+(PGK1p-ARO4 K229L-CYC1t) X-4::(CYC1t-ARO1-TPI1p)+(TDH3p-ARO2-ADH1t)+(TDH2t-ARO3-TEF1p) X-2::(GPM1p-PHA2-CYC1t) XII-5::(GPM1p-AtPAL2-FBA1t) XII-4::(CYC1t-PhCNL-TEF1p)+(PGK1p-RsCHS-ADH1t)+(TDH2t-PsCHI2-TDH3p)	CA02	This study
**PIN08dd**	MATa ura3-52 can1Δ::cas9-natNT2 TRP1 LEU2 HIS3 X-3::(TPI1p-EcaroL-pYX212t)+(ADH1t-ARO7 G141S-TEF1p)+(PGK1p-ARO4 K229L-CYC1t) X-4::(CYC1t-ARO1-TPI1p)+(TDH3p-ARO2-ADH1t)+(TDH2t-ARO3-TEF1p) X-2::(GPM1p-PHA2-CYC1t) XII-5::(GPM1p-AtPAL2-FBA1t) XII-4::(CYC1t-PhCNL-TEF1p)+(PGK1p-RsCHS-ADH1t)+(TDH2t-SbCHI2-TDH3p), ΔPAD1, ΔFDC1	PIN08	This study
**PIN10**	MATa ura3-52 can1Δ::cas9-natNT2 TRP1 LEU2 HIS3 X-3::(TPI1p-EcaroL-pYX212t)+(ADH1t-ARO7 G141S-TEF1p)+(PGK1p-ARO4 K229L-CYC1t) X-4::(CYC1t-ARO1-TPI1p)+(TDH3p-ARO2-ADH1t)+(TDH2t-ARO3-TEF1p) X-2::(GPM1p-PHA2-CYC1t) XII-5::(GPM1p-AtPAL2-FBA1t) XII-4::(CYC1t-PhCNL-TEF1p)+(PGK1p-RsCHS-ADH1t)+(TDH2t-SbCHI2-TDH3p) XII-1::(CYC1t-PhCNL-TEF1p), , ΔPAD1, ΔFDC1	PIN08dd	This study
**PIN11**	MATa ura3-52 can1Δ::cas9-natNT2 TRP1 LEU2 HIS3 X-3::(TPI1p-EcaroL-pYX212t)+(ADH1t-ARO7 G141S-TEF1p)+(PGK1p-ARO4 K229L-CYC1t) X-4::(CYC1t-ARO1-TPI1p)+(TDH3p-ARO2-ADH1t)+(TDH2t-ARO3-TEF1p) X-2::(GPM1p-PHA2-CYC1t) XII-5::(GPM1p-AtPAL2-FBA1t) XII-4::(CYC1t-PhCNL-TEF1p)+(PGK1p-RsCHS-ADH1t)+(TDH2t-SbCHI2-TDH3p) XII-1::(PGK1p-RsCHS-ADH1t), ΔPAD1, ΔFDC1	PIN08dd	This study
**PIN12**	MATa ura3-52 can1Δ::cas9-natNT2 TRP1 LEU2 HIS3 X-3::(TPI1p-EcaroL-pYX212t)+(ADH1t-ARO7 G141S-TEF1p)+(PGK1p-ARO4 K229L-CYC1t) X-4::(CYC1t-ARO1-TPI1p)+(TDH3p-ARO2-ADH1t)+(TDH2t-ARO3-TEF1p) X-2::(GPM1p-PHA2-CYC1t)	PIN08dd	This study

	XII-5::(GPM1p-AtPAL2-FBA1t) XII-4::(CYC1t-PhCNL-TEF1p)+(PGK1p-RsCHS-ADH1t)+(TDH2t-SbCHI2-TDH3p) XII-1::(TDH2t-SbCHI2-TDH3p), ΔPAD1, ΔFDC1		
**PIN13**	MATa ura3-52 can1Δ::cas9-natNT2 TRP1 LEU2 HIS3 X-3::(TPI1p-EcaroL-pYX212t)+(ADH1t-ARO7 G141S-TEF1p)+(PGK1p-ARO4 K229L-CYC1t) X-4::(CYC1t-ARO1-TPI1p)+(TDH3p-ARO2-ADH1t)+(TDH2t-ARO3-TEF1p) X-2::(GPM1p-PHA2-CYC1t) XII-5::(GPM1p-AtPAL2-FBA1t) XII-4::(CYC1t-PhCNL-TEF1p)+(PGK1p-RsCHS-ADH1t)+(TDH2t-SbCHI2-TDH3p) XII-1::(PGK1p-RsCHS-ADH1t)+(TDH2t-SbCHI2-TDH3p), ΔPAD1, ΔFDC1	PIN08dd	This study
**PIN14**	MATa ura3-52 can1Δ::cas9-natNT2 TRP1 LEU2 HIS3 X-3::(TPI1p-EcaroL-pYX212t)+(ADH1t-ARO7 G141S-TEF1p)+(PGK1p-ARO4 K229L-CYC1t) X-4::(CYC1t-ARO1-TPI1p)+(TDH3p-ARO2-ADH1t)+(TDH2t-ARO3-TEF1p) X-2::(GPM1p-PHA2-CYC1t) XII-5::(GPM1p-AtPAL2-FBA1t) XII-4::(CYC1t-PhCNL-TEF1p)+(PGK1p-RsCHS-ADH1t)+(TDH2t-SbCHI2-TDH3p) XII-1::(CYC1t-PhCNL-TEF1p)+(PGK1p-RsCHS-ADH1t)+(TDH2t-SbCHI2-TDH3p), ΔPAD1, ΔFDC1	PIN08dd	This study
**PIN15**	MATa ura3-52 can1Δ::cas9-natNT2 TRP1 LEU2 HIS3 X-3::(TPI1p-EcaroL-pYX212t)+(ADH1t-ARO7 G141S-TEF1p)+(PGK1p-ARO4 K229L-CYC1t) X-4::(CYC1t-ARO1-TPI1p)+(TDH3p-ARO2-ADH1t)+(TDH2t-ARO3-TEF1p) X-2::(GPM1p-PHA2-CYC1t) XII-5::(GPM1p-AtPAL2-FBA1t) XII-4::(CYC1t-PhCNL-TEF1p)+(PGK1p-RsCHS-ADH1t)+(TDH2t-SbCHI2-TDH3p) XII-1::(CYC1t-PhCNL-TEF1p)+(PGK1p-RsCHS-ADH1t)+(TDH2t-SbCHI2-TDH3p) XI-1::(pYX212t-SbCHI2-PGK1p)+(TEF1p-RsCHS-FBA1t)+(CYC1t-PhCNL-TPI1p), ΔPAD1, ΔFDC1	PIN14	This study
**PIN16**	MATa ura3-52 can1Δ::cas9-natNT2 TRP1 LEU2 HIS3 X-3::(TPI1p-EcaroL-pYX212t)+(ADH1t-ARO7 G141S-TEF1p)+(PGK1p-ARO4 K229L-CYC1t) X-4::(CYC1t-ARO1-TPI1p)+(TDH3p-ARO2-ADH1t)+(TDH2t-ARO3-TEF1p) X-2::(GPM1p-PHA2-CYC1t) XII-5::(GPM1p-AtPAL2-FBA1t) XII-4::(CYC1t-PhCNL-TEF1p)+(PGK1p-RsCHS-ADH1t)+(TDH2t-SbCHI2-TDH3p) XII-1::(CYC1t-PhCNL-TEF1p)+(PGK1p-RsCHS-ADH1t)+(TDH2t-SbCHI2-TDH3p) XI-1::(pYX212t-SbCHI2-PGK1p)+(TEF1p-RsCHS-FBA1t)+(CYC1t-PhCNL-TPI1p) X1-3::(pYX212t-SbCHI2-PGK1p)+(TEF1p-RsCHS-FBA1t), ΔPAD1, ΔFDC1	PIN15	This study
**PIN21**	MATa ura3-52 can1Δ::cas9-natNT2 TRP1 LEU2 HIS3 X-3::(TPI1p-EcaroL-pYX212t)+(ADH1t-ARO7 G141S-TEF1p)+(PGK1p-ARO4 K229L-CYC1t) X-4::(CYC1t-ARO1-TPI1p)+(TDH3p-ARO2-ADH1t)+(TDH2t-ARO3-TEF1p) X-2::(GPM1p-PHA2-CYC1t) XII-5::(GPM1p-AtPAL2-FBA1t) XII-4::(CYC1t-PhCNL-TEF1p)+(PGK1p-RsCHS-ADH1t)+(TDH2t-SbCHI2-TDH3p) XII-1::(CYC1t-PhCNL-TEF1p)+(PGK1p-RsCHS-ADH1t)+(TDH2t-SbCHI2-TDH3p) XI-1::(pYX212t-SbCHI2-PGK1p)+(TEF1p-RsCHS-FBA1t)+(CYC1t-PhCNL-TPI1p) X1-3::(pYX212t-SbCHI2-PGK1p)+(TEF1p-RsCHS-FBA1t), ΔPAD1, ΔFDC1,XII-3::(TDH2t-matB-TDH3p)+(tHXT7p-matC-CYC1t)	PIN16	This study
**PIN25**	MATa ura3-52 can1Δ::cas9-natNT2 TRP1 LEU2 HIS3 X-3::(TPI1p-EcaroL-pYX212t)+(ADH1t-ARO7 G141S-TEF1p)+(PGK1p-ARO4 K229L-CYC1t) X-4::(CYC1t-ARO1-TPI1p)+(TDH3p-ARO2-ADH1t)+(TDH2t-ARO3-TEF1p) X-2::(GPM1p-PHA2-CYC1t) XII-5::(GPM1p-AtPAL2-FBA1t) XII-4::(CYC1t-PhCNL-TEF1p)+(PGK1p-RsCHS-ADH1t)+(TDH2t-SbCHI2-TDH3p) XII-1::(CYC1t-PhCNL-TEF1p)+(PGK1p-RsCHS-ADH1t)+(TDH2t-SbCHI2-TDH3p) XI-1::(pYX212t-SbCHI2-PGK1p)+(TEF1p-RsCHS-FBA1t)+(CYC1t-PhCNL-TPI1p) X1-3::(pYX212t-SbCHI2-PGK1p)+(TEF1p-RsCHS-FBA1t), ΔPAD1, ΔFDC1,ΔTSC13 substitued in the ORF of TSC13 ::(MdECR-Fab1t)	PIN16	This study
**PIN33**	MATa ura3-52 can1Δ::cas9-natNT2 TRP1 LEU2 HIS3 X-3::(TPI1p-EcaroL-pYX212t)+(ADH1t-ARO7 G141S-TEF1p)+(PGK1p-ARO4 K229L-CYC1t) X-4::(CYC1t-ARO1-TPI1p)+(TDH3p-ARO2-ADH1t)+(TDH2t-ARO3-TEF1p) X-2::(GPM1p-PHA2-CYC1t) XII-5::(GPM1p-AtPAL2-FBA1t) XII-4::(CYC1t-PhCNL-TEF1p)+(PGK1p-RsCHS-ADH1t)+(TDH2t-SbCHI2-TDH3p) XII-1::(CYC1t-PhCNL-TEF1p)+(PGK1p-RsCHS-ADH1t)+(TDH2t-SbCHI2-TDH3p) XI-1::(pYX212t-SbCHI2-PGK1p)+(TEF1p-RsCHS-	PIN16	This study

	FBA1t)+(CYC1t-PhCNL-TPI1p) X1-3::(pYX212t-SbCHI2-PGK1p)+(TEF1p-RsCHS-FBA1t), V FgF7::(pTPI-mAcc1**-TDH2t), ΔPAD1, ΔFDC1		
**PIN36**	MATa ura3-52 can1Δ::cas9-natNT2 TRP1 LEU2 HIS3 X-3::(TPI1p-EcaroL-pYX212t)+(ADH1t-ARO7 G141S-TEF1p)+(PGK1p-ARO4 K229L-CYC1t) X-4::(CYC1t-ARO1-TPI1p)+(TDH3p-ARO2-ADH1t)+(TDH2t-ARO3-TEF1p) X-2::(GPM1p-PHA2-CYC1t) XII-5::(GPM1p-AtPAL2-FBA1t) XII-4::(CYC1t-PhCNL-TEF1p)+(PGK1p-RsCHS-ADH1t)+(TDH2t-SbCHI2-TDH3p) XII-1::(CYC1t-PhCNL-TEF1p)+(PGK1p-RsCHS-ADH1t)+(TDH2t-SbCHI2-TDH3p) XI-1::(pYX212t-SbCHI2-PGK1p)+(TEF1p-RsCHS-FBA1t)+(CYC1t-PhCNL-TPI1p) X1-3::(pYX212t-SbCHI2-PGK1p)+(TEF1p-RsCHS-FBA1t), ΔPAD1, ΔFDC1,ΔTSC13 substitued in the ORF of TSC13 ::(MdECR-Fab1t), VFgF7::(pTPI-mAcc1**-TDH2t)	PIN25	This study
**PIN37**	MATa ura3-52 can1Δ::cas9-natNT2 TRP1 LEU2 HIS3 X-3::(TPI1p-EcaroL-pYX212t)+(ADH1t-ARO7 G141S-TEF1p)+(PGK1p-ARO4 K229L-CYC1t) X-4::(CYC1t-ARO1-TPI1p)+(TDH3p-ARO2-ADH1t)+(TDH2t-ARO3-TEF1p) X-2::(GPM1p-PHA2-CYC1t) XII-5::(GPM1p-AtPAL2-FBA1t) XII-4::(CYC1t-PhCNL-TEF1p)+(PGK1p-RsCHS-ADH1t)+(TDH2t-SbCHI2-TDH3p) XII-1::(CYC1t-PhCNL-TEF1p)+(PGK1p-RsCHS-ADH1t)+(TDH2t-SbCHI2-TDH3p) XI-1::(pYX212t-SbCHI2-PGK1p)+(TEF1p-RsCHS-FBA1t)+(CYC1t-PhCNL-TPI1p) X1-3::(pYX212t-SbCHI2-PGK1p)+(TEF1p-RsCHS-FBA1t) XI-2::(GPM1p-FNSI-tPS1t), ΔPAD1, ΔFDC1,	PIN16	This study
**CHRY2**	MATa ura3-52 can1Δ::cas9-natNT2 TRP1 LEU2 HIS3 X-3::(TPI1p-EcaroL-pYX212t)+(ADH1t-ARO7 G141S-TEF1p)+(PGK1p-ARO4 K229L-CYC1t) X-4::(CYC1t-ARO1-TPI1p)+(TDH3p-ARO2-ADH1t)+(TDH2t-ARO3-TEF1p) X-2::(GPM1p-PHA2-CYC1t) XII-5::(GPM1p-AtPAL2-FBA1t) XII-4::(CYC1t-PhCNL-TEF1p)+(PGK1p-RsCHS-ADH1t)+(TDH2t-SbCHI2-TDH3p) XII-1::(CYC1t-PhCNL-TEF1p)+(PGK1p-RsCHS-ADH1t)+(TDH2t-SbCHI2-TDH3p) XI-1::(pYX212t-SbCHI2-PGK1p)+(TEF1p-RsCHS-FBA1t)+(CYC1t-PhCNL-TPI1p) X1-3::(pYX212t-SbCHI2-PGK1p)+(TEF1p-RsCHS-FBA1t) XI-2::(GPM1p-FNSII-tPS1t) + (IDP1t-ATR2-tHXT7p), ΔPAD1, ΔFDC1,	PIN16	This study
**PIN38**	MATa ura3-52 can1Δ::cas9-natNT2 TRP1 LEU2 HIS3 X-3::(TPI1p-EcaroL-pYX212t)+(ADH1t-ARO7 G141S-TEF1p)+(PGK1p-ARO4 K229L-CYC1t) X-4::(CYC1t-ARO1-TPI1p)+(TDH3p-ARO2-ADH1t)+(TDH2t-ARO3-TEF1p) X-2::(GPM1p-PHA2-CYC1t) XII-5::(GPM1p-AtPAL2-FBA1t) XII-4::(CYC1t-PhCNL-TEF1p)+(PGK1p-RsCHS-ADH1t)+(TDH2t-SbCHI2-TDH3p) XII-1::(CYC1t-PhCNL-TEF1p)+(PGK1p-RsCHS-ADH1t)+(TDH2t-SbCHI2-TDH3p) XI-1::(pYX212t-SbCHI2-PGK1p)+(TEF1p-RsCHS-FBA1t)+(CYC1t-PhCNL-TPI1p) X1-3::(pYX212t-SbCHI2-PGK1p)+(TEF1p-RsCHS-FBA1t), ΔPAD1, ΔFDC1,ΔTSC13 substitued in the ORF of TSC13 ::(MdECR-Fab1t), VFgF7::(pTPI-mAcc1**-TDH2t), XI-2::(GPM1p-FNSI-tPS1t)	PIN36	This study

Supplementary Table 2. Primers used in this study. [a] Short overlapping regions are indicated in brackets. Red font represent the DNA sequence of the overlapping regions.

Primer name	Sequence 5'-> 3'
**Cinnamic acid production**	
XII-5 up -F	GTAGTGATCATTGGCTTAACG
XII-5 up -R (with GPM1p -F)[a]	GCTCACAAATCTTAAAGTCATACATTGCACGACTAGTGACAATAAATTCAAACCGGT

GPM1p -F	TAGTCGTGCAATGTATGACTTTAAGA
GPM1p -R	CATTGTTTTTATTGTAATATGTGTGTTTGTT
AtPAL2-F (with GPM1p -R)	TAATCCAAACAAACACACATATTACAATAAAAACAATGGATCAAATCGAAGCTATGTTG
AtPAL2-R (with FBA1t -F)	TCATTAAAAAACTATATCAATTAATTTGAATTAACTCAGCAGATAGGAATAGGAGC
FBA1t -F	GTTAATTCAAATTAATTGATATAGTTTTTT
FBA1t -R	AGTAAGCTACTATGAAAGACTTTACAAAG
XII-5 down -F (with FBA1t -R)	CGAGTTCTTTGTAAAGTCTTTCATAGTAGCTTACTCAACTCAGAAGTTTGACAGC
XII-5 down -R	CTCTTTTGCCTTTCAAAAAAG
RtPAL2-F (with GPM1p -R)	CCAAACAAACACACATATTACAATAAAAACAATGGCACCAAGACCAACTTC
RtPAL2-R (with FBA1t -F)	CTCATTAAAAAACTATATCAATTAATTTGAATTAACTTAAGCTAACATTTTCAATAAAACATTATTAATTC
CYC1t-R	GGGTACCGGCCGCAAATTAA
CYC1t-F	GATACCGTCGACCTCGAGTC
AtPAL2-R (with CYC1t-F)	CATAACTAATTACATGACTCGAGGTCGACGGTATCTCAGCAGATAGGAATAGGAGC
AtPAL2-F (with TPIp-R)	TCTATAACTACAAAAAACACATACATAAACTAAAAATGGATCAAATCGAAGCTATGTTG
TPIp-R	CATTTTTAGTTTATGTATGTGTTTTTTGTAG
TPIp-F	GTTTAAAGATTACGGATATTTAACTTAC
XII-5 down -F (with TPIp-F)	ATTCTAAGTAAGTTAAATATCCGTAATCTTTAAACCAACTCAGAAGTTTGACAGC
**For pinocembrin enzymes screening**	
X-II 4 up -F	GTATCCGGCTGTTCCTTCATAG
X-II 4 up -R (with CYC1t-R)	GGACGCTCGAAGGCTTTAATTTGCGGCCGGTACCCTGCCATAGTATGTGTGATGGAAA
ScCCL-R (with CYC1t-F)	CATAACTAATTACATGACTCGAGGTCGACGGTATCTTATCTTGGTTCTCTCAATTGTC
ScCCL-F (with TEFp-R)	AAGCATAGCAATCTAATCTAAGTTTTAATTACAAAATGTTTAGATCTGAATATGCAGATG
TEFp-R	CATTTTGTAATTAAAACTTAGATTAGATTGC
PGKp-R	TTTGTTATATTTGTTGTAAAAAGTAGATAA
PhCHS-F (with PGKp-R)	AGTAATTATCTACTTTTTACAACAAATATAACAAAATGGTTACAGTTGAAGAATACAG
PhCHS-R (with ADH1t-F)	TTAATAATAAAAATCATAAATCATAAGAAATTCGCTTAAGTAGCAACAGAATGTAAAAC
ADH1t-F	GCGAATTTCTTATGATTTATGATTTTT

TDH2t-F	ATTTAACTCCTTAAGTTACTTTAATGATTTAG
MsCHI-R (with TDH2t-F)	AAACTAAATCATTAAAGTAACTTAAGGAGTTAAATTTAATTACCAATTTTAAAAGCACCTTC
MsCHI-F (with TDH3p-R)	ACTTAGTTTCGAATAAACACACATAAACAAACAAAATGGCTGCATCTATCACTGC
TDH3p-R	CATTTTGTTTGTTTATGTGTGTTTATTCGA
TDH3p-F	TCGAGTTTATCATTATCAATACTGCC
X-II 4 down -F (with TDH3p-F)	CTTTGAAATGGCAGTATTGATAATGATAAACTCGAATTCCCCATTAGAGTCAAATAAAAG
XII-4 down -R	TTTCTGCTGTACCTGGATGGTC
PhCNL-R (with CYC1t-F)	CATAACTAATTACATGACTCGAGGTCGACGGTATCTTACAATCTAGCTGGCAAATC
PhCNL-F (with TEFp-R)	CATAGCAATCTAATCTAAGTTTTAATTACAAAATGGATGAATTACCAAAATGTG
Pc4CL2-R (with CYC1t-F)	CATAACTAATTACATGACTCGAGGTCGACGGTATCTTATTTTGGCAAGTCACCTG
Pc4CL2-F (with TEFp-R)	CATAGCAATCTAATCTAAGTTTTAATTACAAAATGGGTGACTGTGTTGCTC
SbCHS2-F (with PGKp-R)	GTAATTATCTACTTTTTACAACAAATATAACAAAATGGTTACAGTTGAAGAATTTCATAG
SbCHS2-R (with ADH1t-F)	CTTATTTAATAATAAAAATCATAAATCATAAGAAATTCGCTTAATTTAATGGAACAGAATGC
RsCHS-F (with PGKp-R)	AGTAATTATCTACTTTTTACAACAAATATAACAAAATGGTTACTGTTGAAGATGTTAG
RsCHS-R (with ADH1t-F)	TTAATAATAAAAATCATAAATCATAAGAAATTCGCTTAAGTACACAATGAATGCAAAAC
SbCHI2-R (with TDH2t-F)	CTAAATCATTAAAGTAACTTAAGGAGTTAAATTTAAAACAATTCAGACAATCTTG
SbCHI2-F (with TDH3p-R)	GTTTCGAATAAACACACATAAACAAACAAAATGTCTGCTTCACCATCTGTTAC
PsCHI-R (with TDH2t-F)	AAACTAAATCATTAAAGTAACTTAAGGAGTTAAATTTATTTTAACAATTCAGAAATTCTTGC
PsCHI-F (with TDH3p-R)	ACTTAGTTTCGAATAAACACACATAAACAAACAAAATGGCAAAACCACCATCTG
**For pinocembrin pathway multicopy**	
XII-1 up -F	GTTGAGCTCTGTCCTTCATGG
XII-1 up -R (with CYC1t-R)	GGACGCTCGAAGGCTTTAATTTGCGGCCGGTACCCGAAAGAACCGAACCGATGC
PhCNL-R (with CYC1t-F)	CATAACTAATTACATGACTCGAGGTCGACGGTATCTTACAATCTAGCTGGCAAATC
PhCNL-F (with TEFp-F)	CATAGCAATCTAATCTAAGTTTTAATTACAAAATGGATGAATTACCAAAATGTG
TEF1p-F	CATTTTGTAATTAAAACTTAGATTAGATTGC
TEF1p-R	ATAGCTTCAAAATGTTTCTACTCCT
XII-1 down -F (with TEFp-R)	AGAGTAAAAAAGGAGTAGAAACATTTTGAAGCTATGTTCAGTTTAGTGCTCTGTCTGAG

XII-1 down -R	TGATGACTGTTTCTCAATCTTTATG
XII-1 up -R (with PGKp-F)	ATGCCTATTGTGCAGATGTTATAATATCTGTGCGTGAAAGAACCGAACCGATGC
PGKp-F	ACGCACAGATATTATAACATCTGC
PGKp-R	TTTGTTATATTTGTTGTAAAAAGTAGATAA
RsCHS-F (with PGKp-R)	AGTAATTATCTACTTTTTACAACAAATATAACAAAATGGTTACTGTTGAAGATGTTAG
RsCHS-R (with ADH1t-F)	TTAATAATAAAAATCATAAATCATAAGAAATTCGCTTAAGTACACAATGAATGCAAAAC
ADH1t-F	GCGAATTTCTTATGATTTATGATTTTT
ADH1t-R	GCATATCTACAATTGGGTGAAATGG
XII-1 down -F (with ADH1t-R)	AAATCGCTCCCCATTTCACCCAATTGTAGATATGCGTTCAGTTTAGTGCTCTGTCTGAG
XII-1 up -R (with TDH2t-R)	TAAAGCACTTAGTATCACACTAATTGGCTTTTCGCGAAAGAACCGAACCGATGC
TDH2t-R	GCGAAAAGCCAATTAGTGTGATAC
TDH2t-F	ATTTAACTCCTTAAGTTACTTTAATGATTTAG
SbCHI2-R	CTAAATCATTAAAGTAACTTAAGGAGTTAAATTTAAAACAATTCAGACAATCTTG
SbCHI2-F	GTTTCGAATAAACACACATAAACAAACAAAATGTCTGCTTCACCATCTGTTAC
XII-1 down -F (with TDH3p-F)	CTTTGAAATGGCAGTATTGATAATGATAAACTCGAGTTCAGTTTAGTGCTCTGTCTGAG
XI-1 up -F	ATTTGTGTGAAGGAATAGTGACG
XI-1 up -R (with pYX212t-R)	GCTCCCTTTAGGGTTCCGATTTAGTGGTTTACGGCCAATGGGCTTGGTATTCCG
pYX212t-R	GCCGTAAACCACTAAATCGGA
pYX212t-F	TAGGGCCCACAAGCTTACG
SbCHI2-R (with pYX212t-F)	GATACCCGGGTCGACGCGTAAGCTTGTGGGCCCTATTAAAACAATTCAGACAATCTTG
SbCHI2-F (with PGKp-R)	AGTAATTATCTACTTTTTACAACAAATATAACAAAATGTCTGCTTCACCATCTGTTAC
RsCHS-F (with TEFp-R)	AAGCATAGCAATCTAATCTAAGTTTTAATTACAAAATGGTTACTGTTGAAGATGTTAG
RsCHS-R (with FBA1t -F)	TCATTAAAAAACTATATCAATTAATTTGAATTAACTTAAGTACACAATGAATGCAAAAC
PhCNL-R (with CYC1t-F)	CATAACTAATTACATGACTCGAGGTCGACGGTATCTTACAATCTAGCTGGCAAATC
PhCNL-F (with TPIp-R)	TCTATAACTACAAAAAACACATACATAAACTAAAAATGGATGAATTACCAAAATGTG
XI-1 down -F (with TPIp-F)	ATTCTAAGTAAGTTAAATATCCGTAATCTTTAAACTTTCTTGGCATTGGCAAATC
XI-1 down -R	AAGAGCCGAGTCCCCATCAG

XI-3 up -F	AGTTACTTGCTCTATGCGTTTGC
XI-3 up -R (with pYX212t-R)	GCTCCCTTTAGGGTTCCGATTTAGTGGTTTACGGCAATCAGACGCACGCTTGGC
XI-3 down -F (with FBA1t -R)	CGAGTTCTTTGTAAAGTCTTTCATAGTAGCTTACTTTACGTGGATTGAGCCAGCA
XI-3 down -R	TGAGAATCCGGACCAGCAGAT
**For chrysin production**	
XI-2 up -F	TAACTCTTCGTATGAGGATTTTC
XI-2 up -R (with TPIp-F)	ATTCTAAGTAAGTTAAATATCCGTAATCTTTAAACTTCTATGGCACATTTTTCTGTTG
PcFNS I-F (with TPIp-R)	TCTATAACTACAAAAAACACATACATAAACTAAAAATGGCTCCAACTACAATTACTG
PcFNS I-R (with CYC1t-F)	CATAACTAATTACATGACTCGAGGTCGACGGTATCTTATGCCAAATTTTCATCAG
XI-2 down -F (with CYC1t -R)	GGACGCTCGAAGGCTTTAATTTGCGGCCGGTACCCCCACAAGTAAAGCTCGTTGAC
XI-2 down -R	ATGGTTGAAAAGGTTACAGAGG
SbFNSII2-F (with TPIp-R)	TCTATAACTACAAAAAACACATACATAAACTAAAAATGGAAGTTACTTTGAACGTTG
SbFNSII2-R (with CYC1t-F)	CATAACTAATTACATGACTCGAGGTCGACGGTATCTTAATGACCAGAAATAACTCTTTC
CYB5-R (with FBA1t -F)	TCATTAAAAAACTATATCAATTAATTTGAATTAACTTATTCGTTCAACAAATAATAAGC
CYB5-F (TDH3p-R)	ACTTAGTTTCGAATAAACACACATAAACAAACAAAATGCCTAAAGTTTACAGTTACC
tHXT7p-R	CATTTTTTGATTAAAATTAAAAAAACTTTTTG
AtATR2-F (with tHXT7p-R)	CAAAAACAAAAAGTTTTTTTAATTTTAATCAAAAAATGTCCTCCTCTTCTTCATCA
AtATR2-R (with pYX212t-F)	GATACCCGGGTCGACGCGTAAGCTTGTGGGCCCTATCACCAGACATCTCTCAAGTATC
XI-2 down -F (with pYX212t-R)	GCTCCCTTTAGGGTTCCGATTTAGTGGTTTACGGCCCACAAGTAAAGCTCGTTGAC
**TSC13::MdECR**	
Ups_TSC13_Fw	GTGGTTCAATAACGACCTTG
FBA1t_Fw_OHMdECR	TTATATTGCCACCATTTTTGTAAGTTAATTCAAATTAATTGATATAGT
MdECR_RV_OHFBA1t	CTATATCAATTAATTTGAATTAACTTACAAAAATGGTGGCAATATAACCC
FAB1t_RV_OHDWN	CCTTGCATTTGGGCATGTTGCAAACAGGAGGAAGTAAGCTACTATGAAAGAC
DownTSC13_FW_OHFBA1t	GTAAAGTCTTTCATAGTAGCTTACTTCCTCCTGTTTGCAACATGCCC
DwnTSC13_RV	CAAGAAGAAATTTGGTCCGATCATTATTGG
veriTSC13_FW	TCTTCTTTACCTCTAGCAATG

veri_TSC13_RV	CAAATATGGATGCTGTTGATGCC
TSC13UP_FW (no promoter)	CCGCCTCTACCACCGAAGCC
TSC13UP_RV_OHMdECR	TTCTGGATACTACTGTTACTTTCATTTTCAAATTAAATTCAAAATATGTATCTCTCTC
MdECR_FW_OHUPS	GAATTTAATTTGAAAATGAAAGTAACAGTAGTATCC
**matBC overexpression**	
XII-3-UP-F	TGTGCCCCTTAAAATTCATATAC
XII-3-UP-R (with TDH2t-R)	TAAAGCACTTAGTATCACACTAATTGGCTTTTCGCGAATGAGCAGGTACCCCTTA
TDH2t-R	GCGAAAAGCCAATTAGTGTGATAC
TDH2t-F	ATTTAACTCCTTAAGTTACTTTAATGATTTAG
matB-R (with TDH2t-F)	AAACTAAATCATTAAAGTAACTTAAGGAGTTAAATTTAAGTTCTTGTGTACAAATCAGC
matB-F(with TH3p-R)	ACTTAGTTTCGAATAAACACACATAAACAAACAAAATGTCTAATCATTTGTTTGATGCAATG
TH3p-R	CATTTTGTTTGTTTATGTGTGTTTATTCGA
tHXT7p-R	CATTTTTTGATTAAAATTAAAAAAACTTTTTG
matC-F (with tHXT7p-R)	CAAAAACAAAAAGTTTTTTTAATTTTAATCAAAAAATGGGTATCGAATTGTTGTCTATC
matC-R (with CYC1t-F)	CATAACTAATTACATGACTCGAGGTCGACGGTATCTTAAACTAAACCTGGAACAACAAAAAC
CYC1t-F	GATACCGTCGACCTCGAGTC
CYC1t-R	GGGTACCGGCCGCAAATTAA
XII-3-down-F (with CYC1t-R)	GGACGCTCGAAGGCTTTAATTTGCGGCCGGTACCCGCATAGAGCTAATTAGGTTTGAG
XII-3-down-R	GAACTTACAAGCTGATTTTGGT
**Acc1** overexpression**	
V-FgF7-up fw	GAATGTCCGGTAATTCCGTTTG
V-FgF7-up rev	CCCTTAAAAGGCAAATCAATAATC
pTPI-F (with -FgF7-up rev)	TATATTAAGATTATTGATTTGCCTTTTAAGGGGTTTAAAGATTACGGATATTTAACTTAC
pTPI-R (with -mACC1-up-F)	CTGTGGAGAAGACTCGAATAAGCTTTCTTCGCTCATTTTTAGTTTATGTATGTGTTTTTTGTAG
mACC1-up-F	ATGAGCGAAGAAAGCTTATTCG
mACC1-up 500bp-R	CAATAGTGGATTCTCGGAGGC
mACC1-down 500bp-F	ATCGTGAGAGAGAACTATTGCC

mACC1-down-R	TTATTTCAAAGTCTTCAACAATTTTTCTTTATC
tTDH2-F (with mACC1-down-R)	CCGATGATAAAGAAAAATTGTTGAAGACTTTGAAATAAATTTAACTCCTTAAGTTACTTTAATGATTTAG
tTDH2-R (with V-FgF7-down fw	TCCGAACAATTATCAAATTACTCGCCAGTTTCTCAGCGAAAAGCCAATTAGTGTGATAC
V-FgF7-down fw	TGAGAAACTGGCGAGTAATTTG
V-FgF7-down rev	CCTCATTCCGTTTCATATGTTTC

Supplementary Table 3. Codon optimized heterologous gene sequences used in this study

Gene	Sources	GenBank ID (CDS)	Optimized Sequence
**AtPAL2**	*Arabidopsis thaliana*	XM_002877863	ATGGATCAAATCGAAGCTATGTTGTGTGGTGGTGGTGAAAAAACAAAAGTTGCTGTTACTACTAAGAC<br>CTTGGCCGATCCATTGAATTGGGGTTTGGCTGCTGATCAAATGAAGGGTTCTCATTTGGATGAAGTCAA<br>GAAGATGGTCGAAGAATACAGAAGACCAGTTGTTAATTTGGGTGGTGAAACTTTGACTATTGGTCAAG<br>TTGCTGCTATTTCTACTGTTGGTGGTTCTGTTAAGGTTGAATTGGCTGAAACTTCTAGAGCTGGTGTTAA<br>GGCTTCTTCTGATTGGGTTATGGAATCTATGAACAAGGGTACTGATTCTTACGGTGTTACTACAGGTTTT<br>GGTGCTACTTCTCATAGAAGAACTAAGAATGGTACTGCCTTGCAAACCGAATTGATCAGATTTTTGAAC<br>GCCGGTATTTTCGGTAACACCAAAGAAACTTGTCATACCTTGCCACAATCTGCTACTAGAGCTGCTATGT<br>TGGTTAGAGTTAACACTTTGTTGCAAGGTTACTCCGGTATCAGATTCGAAATTTTGGAAGCTATCACCTC<br>CTTGTTGAACCATAACATTTCTCCATCTTTGCCATTGAGAGGTACTATTACTGCTTCTGGTGATTTGGTTC<br>CATTGTCTTATATTGCTGGTTTGTTGACTGGTAGACCAAACTCTAAAGCTACTGGTCCAGATGGTGAATC<br>ATTGACTGCTAAAGAAGCTTTTGAAAAGGCTGGTATCTCTACTGGTTTTTTCGACTTGCAACCTAAAGAA<br>GGTTTGGCTTTGGTTAATGGTACAGCTGTTGGTTCTGGTATGGCTTCTATGGTTTTGTTTGAAGCTAACG<br>TTCAAGCTGTTTTGGCCGAAGTTTTGTCTGCTATTTTTGCTGAAGTTATGTCCGGTAAGCCAGAATTCAC<br>TGATCATTTGACCCATAGATTGAAACATCACCCAGGTCAAATTGAAGCTGCTGCAATTATGGAACATAT<br>CTTGGATGGTTCCTCTTACATGAAGTTGGCTCAAAAAGTTCACGAAATGGACCCATTGCAAAAGCCAAA<br>ACAAGATAGATACGCTTTGAGAACTTCTCCACAATGGTTGGGTCCACAAATAGAAGTTATTAGACAAGC<br>CACCAAGTCCATCGAAAGAGAAATCAATTCTGTTAACGACAACCCATTGATCGACGTCAGTAGAAACAA<br>AGCTATTCATGGTGGTAACTTCCAAGGTACTCCAATTGGTGTTTCTATGGACAACACTAGATTGGCTATT<br>GCTGCCATTGGTAAATTGATGTTCGCTCAATTCTCCGAATTGGTCAACGATTTTTACAACAACGGTTTGC<br>CTTCTAACTTGACCGCTTCTTCTAATCCATCATTGGATTACGGTTTTAAGGGTGCTGAAATTGCTATGGCT

			TCATACTGTTCTGAATTGCAATACTTGGCTAACCCAGTTACCTCTCATGTTCAATCTGCTGAACAACACAA<br>TCAAGACGTTAACTCCTTGGGTTTGATCTCTTCTAGAAAGACTTCTGAAGCCGTTGACATCTTGAAGTTG<br>ATGTCTACTACATTCTTGGTCGGTATTTGCCAAGCTGTTGATTTGAGACATTTGGAAGAAAACTTGAGAC<br>AAACCGTCAAGAACACCGTTTCACAAGTTGCTAAGAAAGTTTTGACCACCGGTATTAACGGTGAATTGC<br>ATCCATCTAGATTCTGCGAAAAGGATTTGTTGAAGGTCGTTGATAGAGAACAAGTTTTCACCTACGTTG<br>ATGATCCATGTTCTGCTACTTATCCATTGATGCAAAGATTGAGACAAGTCATCGTTGATCATGCTTTGTC<br>TAATGGTGAAACCGAAAAGAACGCTGTTACCTCCATTTTCCAAAAGATTGGTGCTTTCGAAGAAGAATT<br>GAAGGCCGTTTTGCCAAAAGAAGTTGAAGCAGCTAGAGCAGCTTACGGTAACGGTACTGCTCCAATTC<br>CAAATAGAATCAAAGAATGCAGATCCTACCCATTATACAGATTCGTTAGAGAAGAATTAGGTACTAAGT<br>TGTTGACCGGTGAAAAGGTTGTTTCTCCAGGTGAAGAATTCGATAAGGTTTTCACTGCTATGTGCGAAG<br>GTAAATTGATCGATCCATTGATGGACTGCTTGAAAGAATGGAATGGTGCTCCTATTCCTATCTGCTGA
**RtPAL**	*Rhodotorula toruloides*	M18261.1	ATGGCACCAAGACCAACTTCTCAATCACAAGCTAGAACATGTCCAACTACACAAGTTACTCAAGTTGAT<br>ATTGTTGAAAAGATGTTGGCTGCACCAACTGATTCAACATTAGAATTGGATGGTTACTCTTTGAATTTGG<br>GTGACGTTGTTTCAGCTGCAAGAAAAGGTAGACCAGTTAGAGTTAAAGATTCTGATGAAATTAGATCA<br>AAGATCGATAAGTCTGTTGAATTTTTGAGATCTCAATTATCTATGTCAGTTTACGGTGTTACTACAGGTT<br>TTGGTGGTTCTGCTGATACTAGAACAGAAGATGCAATTTCATTGCAAAAAGCATTGTTGGAACATCAAT<br>TGTGTGGTGTTTTGCCATCTTCATTTGATTCTTTTAGATTGGGTAGAGGTTTAGAAAATTCATTGCCATT<br>GGAAGTTGTTAGAGGTGCTATGACAATCAGAGTTAATTCATTGACTAGAGGTCATTCTGCAGTTAGATT<br>GGTTGTTTTGGAAGCATTGACAAATTTCTTGAACCATGGTATCACTCCAATTGTTCCATTAAGAGGTACA<br>ATTTCTGCATCAGGTGACTTGTCTCCATTATCATATATTGCTGCAGCTATTTCAGGTCATCCAGATTCTAA<br>AGTTCATGTTGTTCATGAGGGTAAAGAAAAGATTTTGTACGCTAGAGAAGCAATGGCTTTGTTTAATTT<br>GGAACCAGTTGTTTTAGGTCCAAAAGAAGGTTTAGGTTTGGTTAATGGTACTGCAGTTTCTGCTTCAAT<br>GGCAACATTAGCTTTGCATGATGCTCACATGTTGTCTTTGTTGTCTCAATCATTAACTGCTATGACAGTT<br>GAAGCAATGGTTGGTCATGCTGGTTCATTTCATCCATTTTTACATGATGTTACTAGACCACATCCAACAC<br>AAATCGAAGTTGCTGGTAACATCAGAAAATTGTTAGAAGGTTCAAGATTTGCTGTTCATCATGAAGAAG<br>AAGTTAAGGTTAAGGATGATGAAGGTATTTTGAGACAAGATAGATACCCATTAAGAACTTCTCCACAAT<br>GGTTGGGTCCATTAGTTTCAGATTTGATTCATGCACATGCTGTTTTAACTATTGAAGCTGGTCAATCTAC<br>TACAGATAACCCATTGATCGATGTTGAAAATAAGACATCACATCATGGTGGTAATTTTCAAGCAGCTGC<br>AGTTGCAAACACTATGGAAAAGACTAGATTGGGTTTGGCTCAAATCGGTAAATTAAACTTCACTCAATT<br>GACAGAAATGTTAAATGCTGGTATGAATAGAGGTTTGCCATCTTGTTTAGCTGCAGAAGATCCATCTTT

			GTCATACCATTGTAAGGGTTTAGATATTGCTGCAGCTGCATACACTTCTGAATTGGGTCATTTGGCTAAT<br>CCAGTTACTACACATGTTCAACCAGCAGAAATGGCTAACCAAGCAGTTAATTCATTAGCTTTGATTTCTG<br>CAAGAAGAACTACAGAATCAAACGATGTTTTGTCTTTGTTATTGGCTACTCATTTGTACTGTGTTTTGCA<br>AGCAATCGATTTGAGAGCTATCGAATTTGAGTTTAAGAAACAATTTGGTCCAGCAATCGTTTCTTTGATC<br>GATCAACATTTTGGTTCAGCTATGACAGGTTCTAATTTGAGAGATGAATTGGTTGAAAAGGTTAATAAG<br>ACTTTGGCAAAGAGATTAGAACAAACAAATTCTTACGATTTGGTTCCAAGATGGCATGATGCTTTTTCAT<br>TTGCTGCAGGTACTGTTGTTGAAGTTTTATCTTCAACATCTTTATCATTGGCTGCAGTTAATGCATGGAA<br>AGTTGCTGCAGCTGAATCTGCTATTTCATTAACTAGACAAGTTAGAGAAACATTTTGGTCTGCAGCTTCA<br>ACTTCTTCACCAGCTTTGTCTTATTTGTCACCAAGAACACAAATCTTGTACGCATTCGTTAGAGAAGAAT<br>TAGGTGTTAAAGCTAGAAGAGGTGACGTTTTCTTGGGTAAACAAGAAGTTACTATCGGTTCTAACGTTT<br>CAAAGATCTATGAAGCAATTAAATCTGGTAGAATTAATAATGTTTTATTGAAAATGTTAGCTTAA
**PhCNL**	*Petunia hybrida*	JN120848.1	ATGGATGAATTACCAAAATGTGGTGCTAACTACGTTCCATTGACTCCATTAACATTTTTGACTAGAGCTT<br>TTAAATCTTACGCAAACAGAACATCAATTATCTATGCTGGTGCAAGATTCACTTGGGAACAAACTTACAA<br>GAGATGTTGTAGATTGGCTTCTTCATTGCAATCTTTGAACATCGTTAAAAATGATGTTGTTTCAGTTTTG<br>GCTCCAAATGTTCCAGCAACTTACGAAATGCATTTTGCAGTTCCAATGGCTGGTGCAGTTTTGAACACAA<br>TTAATACTAGATTGGATCCAATGAACATCGCTATCATCTTGAAGCATTCTGAAGCAAAATTGTTATTTGT<br>TGATTATGAATACTTAGAAAAGGCTAGAAAGGCATTGGAATTGTTGATGTCAACAAACTTCATTACTGC<br>TCAAAATTCTAAGAAAATTTCAATGCCACAAGTTATTTTGATCGATGATTTGTACTCTCCAACAAGAATC<br>CAACAACAAGATCAATTGGAATATGAACAATTGGTTCATCAAGGTAATCCAGAATACGCTCCAGAAAAT<br>ATTGTTGATGATGAATGGGATCCAATCGTTTTGAACTACACTTCTGGTACTACATCAGAACCAAAAGGT<br>GTTGTTTACTCTCATAGAGGTGCATTTTTATCAACATTGAACACTATCATGGGTTGGGAAATGGGTACA<br>GAACCAGTTTATTTGTGGTCTTTGCCAATGTTCCATATCAACGGTTGGACTTTGACATGGGGTATTGCTG<br>CAAGAGGTGGTACTAACGTTTGTATCAGAAACACTACAGCTCAAGAAATCTATTCAAACATCACATTGC<br>ATAAAGTTACTCATATGTGTTGTGCTCCAACAGTTTTTAATATCTTGTTGGAAGCAAAACCACATGAAAG<br>AAGAGAAATTACTACACCAGTTCAAGTTATGGTTGGTGGTGCTCCACCACCAACTACATTGATCGGTAA<br>AATCGAAGAATTGGGTTTCCATGTTGTTCATTGTTACGGTATCACTGAAGCAGGTGGTACTACATTAGTT<br>TGTGAATGGCAATCTGAATGGAATAAGTTGTCAAGAGAAGATCAAGCTAATTTGAAGGCAAGACAAGG<br>TATTTCTGTTTTGGCTTTGGAAGATGTTGATGTTAAAAATTCTAAAACAATGCAATCAGTTCCACATAAC<br>GGTAAAACTATGGGTGAAATCTGTTTGAGAGGTTCTTCAATCATGAAGGGTTACTTCAAGAACGATAAA<br>GCTAACTCTCAAGTTTTTAAAAACGGTTGGTTTTTAACAGGTGACGTTGCAGTTATTCATCAAGATGGTT

			ACTTGGAAATTAAAGATCGTTGTAAGGATATCATCATCTCTGGTGGTGAAAACATCTCTTCAATCGAAG<br>TTGAAAATGCTATTTTAAAACATCCATCAGTTATTGAAGCTGCAGTTGTTGCTATGCCACATCCAAGATG<br>GGGTGAAACACCATGTGCATTCGTTATTAAAACTAAAAATCCAGAAATTAAAGAAGCAGATATTATTGT<br>TCATTGTAAGAAAGAATTGCCAGGTTTTATGGTTCCAAAGCATGTTCAATTCTTGGAAGAATTGCCAAA<br>GACTGGTACTGGTAAAGTTAAGAAATTGCAATTGAGAGAAATGGCTAAGTCTTTCGGTATCTTCGATAA<br>CGCAAATCAAACTTCACAAATTTTGGATTTGCCAGCTAGATTGTAA
**PhCHS**	*Petunia hybrida*	AF233638.1	ATGGTTACAGTTGAAGAATACAGAAAGGCTCAAAGAGCAGAAGGTCCAGCTACTGTTATGGCAATTGG<br>TACTGCTACACCAACTAATTGTGTTGATCAATCTACATACCCAGATTACTACTTCAGAATTACAAATTCAG<br>AACATAAGACTGATTTGAAGGAAAAGTTTAAAAGAATGTGTGAAAAATCTATGATTAAGAAAAGATAT<br>ATGCATTTGACTGAAGAAATTTTAAAAGAAAATCCATCTATGTGTGAATACATGGCACCATCATTGGAT<br>GCTAGACAAGATATTGTTGTTGTTGAAGTTCCAAAATTGGGTAAAGAAGCTGCACAAAAAGCTATTAAA<br>GAATGGGGTCAACCAAAGTCTAAGATCACACATTTGGTTTTCTGTACTACATCAGGTGTTGATATGCCA<br>GGTTGTGATTACCAATTGACTAAATTGTTGGGTTTAAGACCATCAGTTAAGAGATTGATGATGTACCAA<br>CAAGGTTGTTTTGCAGGTGGTACAGTTTTGAGATTAGCAAAGGATTTGGCTGAAAACAATAAGGGTGC<br>AAGAGTTTTGGTTGTTTGTTCTGAAATCACAGCTGTTACTTTTAGAGGTCCAAATGATACTCATTTGGAT<br>TCATTAGTTGGTCAAGCATTGTTTGGTGACGGTGCTGGTGCAATTATTATTGGTTCTGATCCAATTCCAG<br>GTGTTGAAAGACCATTGTTTGAATTAGTTTCTGCTGCACAAACATTGTTACCAGATTCACATGGTGCAAT<br>TGATGGTCATTTGAGAGAAGTTGGTTTAACTTTCCATTTGTTGAAAGATGTTCCAGGTTTAATTTCTAAA<br>AATATCGAAAAATCATTGGAAGAAGCCTTTAGACCTTTGTCTATTTCAGATTGGAACTCTTTGTTTTGGA<br>TTGCACATCCAGGTGGTCCAGCTATTTTAGATCAAGTTGAAATTAAATTGGGTTTAAAGCCAGAAAAAT<br>TGAAGGCAACAAGAAACGTTTTGTCTAACTACGGTAACATGTCTTCAGCTTGTGTTTTGTTTATTTTAGA<br>TGAAATGAGAAAAGCATCTGCAAAAGAAGGTTTAGGTACTACAGGTGAAGGTTTGGAATGGGGTGTTT<br>TATTTGGTTTTGGTCCAGGTTTGACAGTTGAAACTGTTGTTTTACATTCTGTTGCTACTTAA
**SbCHS2**	*Scutellaria baicalensis*	KT963461.1	ATGGTTACAGTTGAAGAATTTCATAGAGCTACTAGAGCAGAAGGTCCAGCTACAGTTTTGGCAATTGGT<br>ACTGCTAATCCACCAAATTGTGTTGAACAATCTACATACGCTGATTACTACTTCAGAATCTGTAAATCAG<br>AACATTTGACTGATTTGAAAAAGAAATTTTCTAGAATGTGTGAAAAATCAGGTATTAAGAAAAGATATA<br>TGCATTTGACAGAAGAATTCTTGAAGGAAAACGATAACTTCACTGCTTACGAAGCACCATCTTTGGATG<br>CAAGACAAGATATCGTTGTTGTTGAAATCCCAAAGTTGGGTAAAGAAGCTGCACAAAAAGCTATTAAA<br>GAATGGGGTCAACCAAAGTCTAAGATCACACATGTTATTTTCTGTACTACATCAGGTGTTGATATGCCA<br>GGTGCAGATTACCAAATCACTAAGTTGTTGGGTTTGAGACCATCAGTTAAAAGATTCATGATGTACCAA

			CAAGGTTGTTTTGCTGGTGGTACAGTTTTGAGAATGGCAAAAGATTTGGCTGAAAATAATGCTGGTGC<br>AAGAGTTTTGGTTGTTTGTTCTGAAATCACAGCAATCACTTTTAGAGGTCCATCTGATACTCATTTGGAT<br>TCATTAGTTGGTCAAGCTTTATTTGGTGACGGTGCTGGTGCAGTTATTGTTGGTTCAGATCCAATTGTTG<br>GTGTTGAAAGACCATTGTTTCAATTAGTTTCTGCTGCACAAACAATTTTACCAGATTCAGAAGGTGCTAT<br>TGATGGTCATGTTAGAGAAGTTGGTTTGACTTTCCATTTGTTGAAAGATGTTCCAGGTTTGATCTCTAAA<br>AATATCGAAAAATCATTGAAAGAAGCTTTTGCACCATTAGGTATCTCTGATTGGAACTCATTGTTTTGGA<br>TTGTTCATCCAGGTGGTCCAGCAATCTTGGATCAAGTTGAAGAAAAGTTGGGTTTGAAGCCAGAAATCA<br>TGGTTCCAACAAGACATGTTTTGTCTGAATACGGTAACATGTCTTCAGCTTGTGTTTTGTTCGTTATGGA<br>TGAAATGAGAAAAGCTTCAGCAAAAGATGGTTGTACTACAACTGGTGAGGGTAAAGATTGGGGTGTTT<br>TGTTTGGTTTTGGTCCAGGTTTAACAGTTGAAACTGTTGTTTTGCATTCTGTTCCATTAAATTAA
**RsCHS**	*Rhododendron simsii*	AJ413277.1	ATGGTTACTGTTGAAGATGTTAGAAGAGCACAAAGAGCTGAAGGTCCAGCAACAGTTATGGCTATTGG<br>TACTGCAACACCATCTAATTGTGTTGATCAATCAACTTACCCAGATTTCTACTTTAGAATTACAAATTCTG<br>AACATAAGGCTGAATTAAAGGAAAAGTTCCAAAGAATGTGTGATAAATCAATGATTAAGAAAAGATAT<br>ATGTACTTAACTGAAGAAATTTTGAAGGAAAACCCATCTGTTTGTGAATATATGGCTCCATCATTAGATG<br>CAAGACAAGATATGGTTGTTGTTGAAGTTCCAAAATTGGGTAAAGAAGCTGCAACTAAAGCTATTAAA<br>GAATGGGGTCAACCAAAGTCTAAGATCACACATTTGGTTTTCTGTACTACATCAGGTGTTGATATGCCA<br>GGTGCAGATTACCAATTGACTAAATTGTTGGGTTTAAGACCATCTGTTAAGAGATTGATGATGTACCAA<br>CAAGGTTGTTTTGCTGGTGGTACAGTTTTGAGATTGGCTAAAGATTTGGCAGAAAACAATAAGGGTGC<br>TAGAGTTTTGGTTGTTTGTTCAGAAATCACTGCTGTTACTTTTAGAGGTCCATCTGATACACATTTGGATT<br>CATTGGTTGGTCAAGCATTGTTTGGTGACGGTGCTGCTGCTATTATTGTTGGTGCAGATCCAGTTCCAG<br>AAGTTGAAAAGCCATTGTTCGAATTGGTTTCTGCTGCTCAAACTATTTTACCAGATTCAGATGGTGCTAT<br>TGATGGTCATTTGAGAGAAGTTGGTTTGACATTCCATTTGTTGAAGGATGTTCCAGGTTTGATCTCTAAA<br>AATATCGAAAAAGCATTGACTGAAGCATTTCAACCATTGGGTATTTCTGATTGGAACTCTATTTTCTGGA<br>TTGCTCATCCAGGTGGTCCAGCAATTTTGGATCAAGTTGAATTGAAATTATCTTTGAAGCCAGAAAAATT<br>GAGAGCTACAAGACATGTTTTGTCAGAATACGGTAACATGTCTTCAGCATGTGTTTTGTTTATTTTGGAT<br>GAAATGAGAAGAAAATCTGCTGAAGAAGGTTTAAAAACTACAGGTGAAGGTTTAGAATGGGGTGTTTT<br>GTTTGGTTTTGGTCCAGGTTTGACTGTTGAAACAGTTGTTTTGCATTCATTGTGTACTTAA
**MsCHI**	*Medicago sativa*	M91079.1	ATGGCTGCATCTATCACTGCAATCACAGTTGAAAATTTGGAATATCCAGCTGTTGTTACTTCTCCAGTTA<br>CTGGTAAATCATACTTTTTAGGTGGTGCAGGTGAAAGAGGTTTGACTATCGAGGGTAACTTCATCAAGT<br>TTACTGCTATTGGTGTTTATTTGGAAGATATTGCTGTTGCATCATTGGCTGCAAAGTGGAAGGGTAAAT

			CTTCAGAAGAATTGTTAGAAACTTTAGATTTCTACAGAGATATTATCTCTGGTCCATTCGAAAAATTGAT<br>CAGAGGTTCAAAGATCAGAGAATTGTCTGGTCCAGAATATTCAAGAAAAGTTATGGAAAACTGTGTTG<br>CACATTTGAAGTCTGTTGGTACTTATGGTGACGCTGAAGCAGAAGCTATGCAAAAATTTGCAGAAGCCT<br>TTAAACCAGTTAATTTTCCACCAGGTGCTTCTGTTTTCTATAGACAATCACCAGATGGTATTTTGGGTTTA<br>TCTTTTTCACCAGATACTTCTATTCCAGAAAAGGAAGCTGCATTGATCGAAAATAAGGCAGTTTCTTCAG<br>CTGTTTTGGAAACAATGATTGGTGAACATGCTGTTTCACCAGATTTGAAAAGATGTTTAGCTGCAAGAT<br>TGCCAGCATTGTTAAATGAAGGTGCTTTTAAAATTGGTAATTAA
**SbCHI2**	*Scutellaria baicalensis*	KT963462.1	ATGTCTGCTTCACCATCTGTTACTAAGGTTTTGGTTGAATCAATCGAATTTTCTCCAGCTGCAAAACCACC<br>AGGTTCTTCAAATACATTGTTTTTAGGTGGTGCTGGTGTTAGAGGCATGGAAATCCAGGGTAACTTCGT<br>TAAGTTTACTGCAATCGGTGTTTATTTGGAAGATTCAGCTGTTCCATCTTTAGCAGTTAATTGGAAAGGT<br>AAAACTGCTGAAGAATTGACAGAATCAGATGATTTCTTTAGAGAAATCGTTTCTGGTCCATTCGAAAAG<br>TTTACTAAGGTTACAATGATCTTGCCATTGACAGGTAAACAATACTCTGAAAAGGTTGCAGAAAACTGT<br>GTTGCTTACTGGAAAGCAGTTGGTAAATACACTGATGCTGAATCAGAAGCAATCGATAAGTTCTTGCAA<br>GTTTTTAAAGATGAAACATTTGCTCCAGGTGCATCTATTTTGTTTACTCAATCACCAGCTGGTTCTTTGAC<br>AATCTCATTTTCTAAGGATGGTTCAATTCCAGAACAGGGTAAAGCTGTTATTGAAAATAAGCAATTGTC<br>AGAAGCAGTTTTGGAATCTATCATCGGTAAACATGGTGTTTCACCATCTGCAAAACAATCATTAGCTGC<br>AAGATTGTCTGAATTGTTTTAA
**PsCHI**	*Paeonia suffruticosa*	GQ984161	ATGGCAAAACCACCATCTGTTTCAGGTGTTAACATCGAATCTTATGCTTTTCCACCAACTGTTAAACCAC<br>CAGGTTCTACTAAAACATTGTTTTTAGGTGGTGCAGGTGTTAGAGGTTTGGAAGTTCCAACTGGTCAAT<br>TCGTTAAGTTTACTGCTATCGGTGTTTACTTGGAAGATAACGCAATCACTTCATTAGCTGTTAAGTGGAA<br>AGGTAAAACTGCTGAAGAATTAACAGAATCTGATGATTTCTTTAGAGATATCGTTACAGGTCCATTCGA<br>AAAGTTTACTCAAGTTACAATGATCTTGCCATTGACTGGTCAACAATACTCAGAAAAGGTTACAGAAAA<br>CTGTGTTGCTTACTGGAAAGCAGTTGGTGCTTACACTGATGCTGAAGCATCTGCTATTGAAAAGTTTATT<br>GAAGTTTTTAAAGATGAAAAGTTTCCACCAGGTTCTTCAATTTTGTTTACTCAAACACCAGAAGGTTCTT<br>TAACAATCGGTTTTTCAAAGGATGGTGTTTTGCCAGAAGTTGGTAATGCAGTTGTTGAAAATAAGCAAT<br>TGTCTGAAGCTGTTTTAGAATCAATCATCGGTAAACATGGTGTTTCTCCAGAAGCTAAACAATCATTAGC<br>TGCAAGAATTTCTGAATTGTTAAAATAA
**SbFNSII-2**	*Scutellaria baicalensis*	KT963454.1	ATGGAAGTTACTTTGAACGTTGCTTTGTTGTTGTTGTCTGCTGCAGTTTGTTTGATGGTTTTTACAGGTA<br>AAAGAAGAAGAAGATTGCCAAATCCACCAGGTCCATTCCCATTGCCATTGATCGGTAATTTGAATTTGG<br>TTTCTCCAAGATTGCATCATACTTTCCATATGTTGGCTCAAAGATACGGTCCAATCATGAAGTTTAGATT

			GGGTTCTATTCCATGTTTGGTTGTTTCAACTCCAGAATTGGCAAAGGATATCTTGAAGACACATGAATTG<br>ATTTTCTCTTCACGTGTTAAGTCTACTGCTATCGATATCGTTACATACGGTGTTTCTTTTGCATTTTCACCA<br>TATGGTCCATACTGGAAGTACATTAAGAAATTGTGTACATACGAATTGTTGGGTTCTAGAATGTTGAAC<br>CATTTCGAACCATTGAGAGCTTTGGAAGTTAGAGAATTCTTGAAGGATGTTATGGCTATGGGTAAAGCT<br>GGTAAATCTTTTAATGTTACTGAAGAATTGATGAAATTAACATCTAATGTTATGTCAAATATGATGTTGT<br>CTATTAGAGCTGCAGAATCAGAAGAACAAGCTGAAGTTGCAAGAACTTTGATCAGAGAAGTTTCTCAAT<br>TGTTCGGTGAATTCGATTTCGGTGACATGTTGTGGTTCTGTAAGTCTTTTGATTTTCAAGGTATTAAGAA<br>AAGATCAAAGGATATCAAAGTTAGATACGATGCATTGTTGGAAAAGATTTTGACAGATAGAGAAAACG<br>TTAGAAGACAAAACGGTGTTGTTGAACCAAAGGATATGTTGGATATGTTCTTGGATATCATGGAAGGT<br>GGTAAAACTGATGTTGAATTCACTAGAGAACATTTGAAGGCTGTTATTTTGGATTTCTTGACTGCTGGTA<br>CAGATACTACAGCAATTACTGTTGAATGGGTTTTGGCAGAATTGATGAACTCTCCAAAGGCTATGAAGA<br>AAGCACAAGATGAAATGGATAGAGTTGTTGGTAGAGAAAGAATGATGGCTGAATCAGATGCACCAAA<br>TTTGCCATACTTTTTGGCTATTATTAAGGAAACTTTTAGATTACATCCACCAATCCCATTGATCATCAGAA<br>GATCTATCGAAGATTGTGTTATCGATGGTTACCATATTCCAGCTGATACATTGGCTTTTATTAACGTTTG<br>GTCAATGGGTAGAAACGAAAAGTACTGGGATTCTCCATTATCTTTTAGACCAGAAAGATTTTTGGAAGG<br>TGACAATGCTGCAATCGATATCAAGGGTATGCATTTCGAATTGTTACCATTTGGTTCTGGTAGAAGAGG<br>TTGTCCAGGCATGTTGTCAGCTATCCAAGAAGTTTTGATCATCGCAGGTACAGTTATTCAATGTTTCGAT<br>TGGGAACAAGCTGATGGTTCTGGTAGAGTTGATATGTCAGAAAGACCAGGTTTAACTACACCAAGAGA<br>AATTGATTTGGTTTGTAGAGTTGTTCCAAGAGTTGATGAAAGAGTTATTTCTGGTCATTAA
**PcFNSI**	*Petroselinum crispum*	AY230247.1	ATGGCTCCAACTACAATTACTGCTTTGGCAAAGGAAAAGACTTTGAATTTGGATTTCGTTAGAGATGAA<br>GATGAAAGACCAAAAGTTGCTTACAACCAATTTTCTAACGAAATCCCAATTATTTCATTGGCAGGTTTAG<br>ATGATGATTCTGATGGTAGAAGACCAGAAATCTGTAGAAAGATCGTTAAAGCATGTGAAGATTGGGGT<br>ATCTTCCAAGTTGTTGATCATGGTATCGATTCTGGTTTGATTTCAGAAATGACAAGATTGTCAAGAGAAT<br>TTTTCGCTTTGCCAGCAGAAGAAAAATTAGAATACGATACTACAGGTGGTAAAAGAGGTGGTTTTACTA<br>TTTCAACAGTTTTGCAAGGTGACGATGCTATGGATTGGAGAGAATTTGTTACTTACTTCTCTTACCCAAT<br>TAATGCAAGAGATTATTCAAGATGGCCAAAGAAACCAGAAGGTTGGAGATCTACTACAGAAGTTTACT<br>CAGAAAAATTGATGGTTTTGGGTGCTAAATTGTTAGAAGTTTTGTCTGAAGCAATGGGTTTGGAAAAG<br>GGTGACTTGACAAAAGCATGTGTTGATATGGAACAAAAGGTTTTGATTAATTACTACCCAACTTGTCCA<br>CAACCAGATTTGACATTAGGTGTTAGAAGACATACTGATCCAGGTACTATCACAATCTTGTTGCAAGAT<br>ATGGTTGGTGGTTTGCAAGCTACTAGAGATGGTGGTAAAACTTGGATCACAGTTCAACCAGTTGAAGG

			TGCATTTGTTGTTAATTTGGGTGACCATGGTCATTATTTGTCAAACGGTAGATTCAGAAATGCTGATCAT<br>CAAGCAGTTGTTAATTCTACATCTTCAAGATTATCAATCGCTACTTTCCAAAATCCAGCTCAAAACGCAA<br>TCGTTTACCCATTGAAGATCAGAGAAGGTGAAAAGGCAATTTTAGATGAAGCTATCACTTATGCTGAAA<br>TGTACAAGAAATGTATGACAAAGCATATCGAAGTTGCTACTAGAAAGAAATTGGCTAAGGAAAAGAGA<br>TTGCAAGATGAAAAGGCTAAATTGGAAATGAAGTCTAAGTCAGCTGATGAAAATTTGGCATAA
**RtmatB**	*Rhizobium trifolii*	AF117694.1	ATGTCTAATCATTTGTTTGATGCAATGAGAGCTGCAGCTCCTGGTAATGCTCCTTTTATTAGAATCGATA<br>ACACTAGAACATGGACTTACGATGATGCATTTGCTTTATCTGGTAGAATTGCATCAGCTATGGATGCATT<br>GGGTATTAGACCAGGTGACAGAGTTGCTGTTCAAGTTGAAAAATCTGCAGAAGCTTTGATCTTGTATTT<br>GGCATGTTTGAGATCAGGTGCTGTTTATTTGCCATTGAATACAGCATACACTTTGGCTGAATTGGATTAC<br>TTCATCGGTGACGCAGAACCAAGATTGGTTGTTGTTGCTTCTTCAGCAAGAGCTGGTGTTGAAACTATT<br>GCAAAACCAAGAGGTGCTATTGTTGAAACATTAGATGCAGCTGGTTCTGGTTCATTGTTAGATTTGGCA<br>AGAGATGAACCAGCTGATTTTGTTGATGCATCTAGATCAGCTGATGATTTGGCAGCTATTTTGTACACTT<br>CTGGTACTACAGGTAGATCAAAAGGTGCAATGTTGACACATGGTAATTTGTTGTCAAACGCTTTGACTT<br>TGAGAGATTTTTGGAGAGTTACAGCAGGTGACAGATTGATCCATGCTTTGCCAATCTTCCATACTCATG<br>GTTTATTCGTTGCTACAAACGTTACTTTGTTAGCAGGTGCTTCTATGTTTTTGTTGTCAAAGTTCGATCCA<br>GAAGAAATCTTGTCTTTGATGCCACAAGCTACTATGTTGATGGGTGTTCCAACATTCTACGTTAGATTGT<br>TGCAATCACCAAGATTGGATAAGCAAGCAGTTGCTAACATCAGATTGTTTATTTCTGGTTCAGCACCATT<br>GTTAGCTGAAACACATACTGAATTTCAAGCAAGAACTGGTCATGCTATTTTAGAAAGATACGGTATGAC<br>AGAAACTAACATGAACACTTCTAACCCATACGAAGGTAAAAGAATTGCTGGTACAGTTGGTTTTCCATT<br>GCCAGATGTTACAGTTAGAGTTACTGATCCAGCAACAGGTTTAGCTTTGCCACCAGAACAAACTGGTAT<br>GATCGAAATTAAAGGTCCAAACGTTTTTAAAGGTTACTGGAGAATGCCAGAAAAGACTGCAGCTGAAT<br>TCACTGCTGATGGTTTCTTTATTTCTGGTGACTTGGGTAAAATCGATAGAGATGGTTACGTTCATATTGT<br>TGGTCGTGGTAAAGATTTGGTTATTTCTGGTGGTTACAACATCTATCCAAAGGAAGTTGAAGGTGAAAT<br>CGATCAAATCGAAGGTGTTGTTGAATCAGCTGTTATTGGTGTTCCACATCCAGATTTTGGTGAAGGTGT<br>TACTGCTGTTGTTGTTAGAAAACCAGGTGCAGCTTTGGATGAAAAGGCAATCGTTTCTGCTTTGCAAGA<br>TAGATTGGCAAGATACAAGCAACCAAAGAGAATCATCTTCGCTGAAGATTTGCCAAGAAATACAATGG<br>GTAAAGTTCAAAAGAATATCTTGAGACAACAATATGCTGATTTGTACACAAGAACTTAA
**RtmatC**	*Rhizobium trifolii*	AF117694.1	ATGGGTATCGAATTGTTGTCTATCGGTTTGTTGATTGCTATGTTCATCATCGCAACTATCCAACCAATTA<br>ATATGGGTGCTTTGGCATTTGCTGGTGCATTTGTTTTAGGTTCTATGATCATCGGTATGAAGACAAACG<br>AAATCTTCGCTGGTTTCCCATCAGATTTGTTTTTGACTTTGGTTGCAGTTACATATTTGTTCGCTATCGCA

			CAAATTAATGGTACTATCGATTGGTTGGTTGAATGTGCTGTTAGATTAGTTAGAGGTAGAATTGGTTTG<br>ATTCCATGGGTTATGTTCTTGGTTGCTGCAATCATCACAGGTTTCGGTGCTTTGGGTCCAGCTGCAGTTG<br>CTATTTTGGCACCAGTTGCTTTGTCTTTCGCAGTTCAATACAGAATCCATCCAGTTATGATGGGTTTAAT<br>GGTTATTCATGGTGCTCAAGCAGGTGGTTTTTCTCCAATCTCAATCTATGGTGGTATCACTAACCAAATC<br>GTTGCTAAAGCAGGTTTACCATTTGCTCCAACATCTTTGTTTTTATCTTCATTTTTCTTTAATTTGGCTATC<br>GCAGTTTTGGTTTTCTTTGTTTTCGGTGGTGCTAGAGTTATGAAACATGATCCAGCATCATTGGGTCCAT<br>TACCAGAATTGCATCCAGAAGGTGTTTCTGCTTCAATTAGAGGTCATGGTGGTACTCCAGCTAAACCAA<br>TTAGAGAACATGCATACGGTACTGCTGCAGATACAGCTACTACATTGAGATTGAACAACGAAAGAATC<br>ACTACATTGATCGGTTTGACAGCTTTAGGTATCGGTGCATTGGTTTTTAAGTTTAATGTTGGTTTAGTTG<br>CAATGACTGTTGCTGTTGTTTTGGCATTGTTATCTCCAAAGACACAAAAGGCTGCAATCGATAAAGTTTC<br>TTGGTCAACTGTTTTGTTGATCGCTGGTATCATCACATACGTTGGTGTTATGGAAAAAGCTGGTACTGTT<br>GATTACGTTGCAAATGGTATTTCTTCATTGGGTATGCCATTGTTGGTTGCTTTGTTGTTGTGTTTCACTGG<br>TGCAATTGTTTCAGCTTTTGCATCTTCAACAGCTTTATTGGGTGCAATCATCCCATTGGCTGTTCCATTTT<br>TATTGCAAGGTCATATCTCTGCTATTGGTGTTGTTGCTGCAATCGCAATCTCAACTACAATCGTTGATAC<br>TTCTCCATTTTCAACAAATGGTGCTTTAGTTGTTGCTAATGCACCAGATGATTCTAGAGAACAAGTTTTG<br>AGACAATTGTTGATCTATTCAGCATTGATCGCTATCATCGGTCCAATTGTTGCTTGGTTGGTTTTTGTTGT<br>TCCAGGTTTAGTTTAA
**MdECR**	*Malus domestica*	XM_008384596.3	ATGAAAGTAACAGTAGTATCCAGAAGTGGTAGAGAAGTAGTCAAGGGTGGTTTAGAATTATCCGACAG<br>TGCAACAGTAGCCGATTTACAAGATGCTATCCATAAGAGAACTAAAAAATTCTATCCAGCAAGACAAAG<br>ATTGACATTACCAGTTCAACCAGGTTCAAAAGAAAGACCAGTTGTTTTGTCTTACAAAAAATCATTACAA<br>GATTACATCTCTGGTAATTCAGATAACTTGACTGTTGTTTTCAAAGATTTAGGTCCACAAGTTTCTTACA<br>GAACATTGTTTTTCTTTGAATACTTGGGTCCATTGATCTTGTACCCAATTTTCTATTACTTCCCAGTTTACG<br>ATTACTTGGGTTTCAAGGGTGATAGAGTTATCCATCCAGTTCAAACTTATGCTTTGTACTACTGGTGTTT<br>CCATTACTTCAAAAGAATTATGGAAACTTTCTTTGTTCATAGATTCTCTCATGCTACATCTCCATTGTCAA<br>ACGTTTTCAGAAACTGTGCATACTACTGGTCATTCGGTGCTTTCATCGCATACTACTTGAACCATCCATTA<br>TACACACCAGTTTCTGATTTACAAATGAAAATTGGTTTTGGTATTGGTATTATTTGTCAAATTTCTAACTT<br>CTACTGTCATATTTTGTTAAGAAATTTGAGATCACCAGATGGTAATGGTGGTTACCAAATCCCAAGAGG<br>TTTCTTGTTCAACATCGTTACTTGTGCAAACTACACTACAGAAATTTACCAATGGTTGGGTTTCAACATTG<br>CTACTCAAACAGTTGCAGGTTACATTTTCTTGATCGTTGCTGCATCAATTATGACAAATTGGGCTTTGGC

			AAAACATAGAAGATTGAAGAAAATTTTTGATGGTAAGGACGGTAGACCTAAGTATCCAAGAAGATGGG<br>TTATATTGCCACCATTTTTGTAA
**AtATR2**	*Arabidopsis thaliana*	NM_119167.4	ATGTCCTCCTCTTCTTCATCATCCACCTCTATGATTGATTTGATGGCCGCTATTATCAAGGGTGAACCAGT<br>TATAGTTTCTGATCCAGCTAATGCTTCTGCCTATGAATCTGTTGCTGCTGAATTATCCTCCATGTTGATCG<br>AAAACAGACAATTCGCTATGATCGTCACTACCTCTATTGCTGTTTTGATTGGTTGCATCGTTATGTTGGTT<br>TGGAGAAGATCTGGTTCTGGTAACTCTAAAAGAGTCGAACCATTGAAGCCATTGGTTATCAAACCTAGA<br>GAAGAAGAAATTGACGACGGTAGAAAGAAGGTTACCATTTTCTTTGGTACTCAAACCGGTACTGCTGA<br>AGGTTTTGCTAAAGCTTTGGGTGAAGAAGCTAAAGCCAGATACGAAAAGACTAGATTCAAGATCGTTG<br>ACTTGGATGATTACGCTGCAGATGATGATGAATACGAAGAAAAGTTGAAGAAAGAAGATGTCGCCTTT<br>TTCTTCTTGGCTACTTATGGTGATGGTGAACCTACTGATAATGCTGCTAGATTTTACAAGTGGTTCACCG<br>AAGGTAATGATAGAGGTGAATGGTTGAAAAACTTGAAGTACGGTGTTTTCGGTTTGGGTAATAGACAA<br>TACGAACACTTCAACAAGGTTGCCAAGGTTGTTGATGATATCTTGGTTGAACAAGGTGCCCAAAGATTG<br>GTTCAAGTTGGTTTAGGTGATGATGACCAATGCATCGAAGATGATTTTACTGCTTGGAGAGAAGCTTTG<br>TGGCCAGAATTGGATACAATCTTGAGAGAAGAAGGTGATACTGCTGTTGCTACTCCATATACTGCTGCT<br>GTTTTAGAATACAGAGTTTCCATCCACGATTCCGAAGATGCTAAGTTCAACGATATTAACATGGCTAAC<br>GGTAACGGTTACACCGTTTTTGATGCTCAACATCCATACAAGGCTAACGTTGCTGTTAAGAGAGAATTG<br>CATACTCCAGAATCTGACAGATCCTGCATTCATTTGGAATTCGATATTGCTGGTTCCGGTTTGACTTACG<br>AAACTGGTGATCATGTTGGTGTTTTGTGCGATAACTTGTCTGAAACTGTTGATGAAGCCTTGAGATTATT<br>GGATATGTCTCCAGATACCTACTTCTCCTTGCATGCCGAAAAAGAAGATGGTACTCCAATCTCTTCATCT<br>TTGCCACCACCATTTCCACCATGTAATTTGAGAACTGCTTTGACCAGATACGCTTGCTTGTTGTCATCTCC<br>AAAAAAGTCTGCTTTGGTTGCTTTGGCTGCTCATGCTTCAGATCCAACTGAAGCTGAAAGATTGAAACA<br>TTTGGCTTCTCCAGCTGGTAAGGATGAATATTCTAAATGGGTTGTTGAATCCCAAAGATCCTTGTTGGA<br>AGTTATGGCTGAATTTCCATCTGCTAAACCACCATTGGGTGTTTTTTTTGCTGGTGTTGCTCCAAGATTG<br>CAACCTAGATTCTACTCTATTTCCTCCTCCCCAAAAATTGCCGAAACCAGAATTCATGTTACTTGCGCTTT<br>GGTCTACGAAAAAATGCCAACTGGTAGAATCCATAAGGGTGTTTGTTCTACCTGGATGAAGAATGCTGT<br>TCCTTACGAAAAGTCCGAAAACTGTTCTTCTGCTCCAATCTTCGTTAGACAATCCAATTTCAAGTTGCCAT<br>CCGATTCTAAGGTTCCAATTATCATGATTGGTCCAGGTACTGGTTTGGCTCCTTTTAGAGGTTTTTTACA<br>AGAAAGATTGGCCTTGGTCGAATCCGGTGTTGAATTGGGTCCATCTGTTTTGTTTTTCGGTTGCAGAAA<br>CAGAAGAATGGACTTCATCTACGAAGAAGAATTACAAAGATTCGTCGAATCAGGTGCTTTGGCAGAAT<br>TGTCAGTTGCTTTTTCTAGAGAAGGTCCAACAAAAGAATACGTCCAACACAAGATGATGGATAAGGCTT

	CTGATATCTGGAACATGATTTCTCAAGGTGCCTACTTGTATGTTTGTGGTGATGCTAAAGGTATGGCCA GAGATGTTCATAGATCCTTGCATACAATTGCCCAAGAACAAGGTTCTATGGACTCTACAAAAGCAGAAG GTTTCGTCAAGAACTTGCAAACTTCTGGTAGATACTTGAGAGATGTCTGGTGA

Supplementary Table 4. Expression cassettes with the corresponding integration sites used in this study.

Integration site	Expression casettes
**X-3**	X-3::(TPI1p-EcaroL-pYX212t)+(ADH1t-ARO7 G141S-TEF1p)+(PGK1p-ARO4 K229L-CYC1t)
**X-4**	X-4::(CYC1t-ARO1-TPI1p)+(TDH3p-ARO2-ADH1t)+(TDH2t-ARO3-TEF1p)
**X-2**	X-2::(GPM1p-PHA2-CYC1t) XII-5::(GPM1p-AtPAL2-FBA1t)
**XII-5**	XII-5::(GPM1p-AtPAL2-FBA1t)
**XII-4**	XII-4::(CYC1t-PhCNL-TEF1p)+(PGK1p-RsCHS-ADH1t)+(TDH2t-SbCHI2-TDH3p)
**XII-1**	XII-1::(CYC1t-PhCNL-TEF1p)+(PGK1p-RsCHS-ADH1t)+(TDH2t-SbCHI2-TDH3p)
**XI-1**	XI-1::(pYX212t-SbCHI2-PGK1p)+(TEF1p-RsCHS-FBA1t)+(CYC1t-PhCNL-TPI1p)
**XI-3**	X1-3::(pYX212t-SbCHI2-PGK1p)+(TEF1p-RsCHS-FBA1t)
**XII-3**	XII-3::(TDH2t-matB-TDH3p)+(tHXT7p-matC-CYC1t)
**V-FgF7**	V-FgF7::(pTPI-mAcc1**-TDH2t)
**XI-2**	XI-2::(GPM1p-FNSII-tPS1t) + (IDP1t-ATR2-tHXT7p)
**XI-2**	XI-2::(GPM1p-FNSI-tPS1t)
**TSC13ORF**	(MdECR-FBA1t)

p indicates promoter, t indicates terminator

Supplementary Table 5. gRNAs used in this study

Plasmid ID	Relevant characteristics	Reference
**pQC010**	2µm ampR KlURA3 gRNA-XII-4.Y	[2]
**pQC005**	2µm ampR KlURA3 gRNA-X-3.Y	[2]
**pQC008**	2µm ampR KlURA3 gRNA-X-4.Y	[2]
**pQC032**	2µm ampR URA3 gRNA-XII-1.Y [2x]	[2]
**pQC033**	2µm ampR URA3 gRNA-XII-5.Y [2x]	[2]
**pQC030**	2µm ampR URA3 gRNA-XI-1.Y	[2]
**pQC133**	2µm ampR URA3 gRNA-XII-3.	[2]
**pQC006**	2µm ampR KlURA3 gRNA-XI-3.Y	[2]
**pQC029**	2µm ampR URA3 gRNA-X-2.Y [2x]	[2]
**pMEL10**	2µm ampR KlURA3 gRNA-CAN1.	[2]
**pMT01**	2µm ampR KlURA3 gRNA-TSC13.	this study, negative strand
**FgF7**	2µm ampR KlURA3 gRNA-V FgF7.	Donated by Xiang Jiao, constrcuted from [3]

References

(1) Mans, R., van Rossum, H. M., Wijsman, M., Backx, A., Kuijpers, N. G. A., van den Broek, M., Daran-Lapujade, P., Pronk, J. T., van Maris, A. J. A., and Daran, J.-M. G. (2015) CRISPR/Cas9: a molecular Swiss army knife for simultaneous introduction of multiple genetic modifications in *Saccharomyces cerevisiae*. *FEMS Yeast Res. 15*.
(2) Liu, Q., Yu, T., Li, X., Chen, Y., Campbell, K., Nielsen, J., and Chen, Y. (2019) Rewiring carbon metabolism in yeast for high level production of aromatic chemicals. *Nat Commun 10*, 4976.
(3) Bourgeois, L., Pyne, M. E., and Martin, V. J. J. (2018) A Highly Characterized Synthetic Landing Pad System for Precise Multicopy Gene Integration in Yeast. *ACS Synth. Biol. 7*, 2675–2685.
